# Care Pathways in Rehabilitation for Children and Adolescents with Cerebral Palsy: Distinctiveness of the Adaptation to the Italian Context

**DOI:** 10.3390/children11070852

**Published:** 2024-07-13

**Authors:** Silvia Faccioli, Silvia Sassi, Emanuela Pagliano, Cristina Maghini, Silvia Perazza, Maria Francesca Siani, Giada Sgherri, Giuseppina Mariagrazia Farella, Maria Foscan, Marta Viganò, Silvia Sghedoni, Arianna Valeria Bai, Giulia Borelli, Adriano Ferrari

**Affiliations:** 1 Paediatric Rehabilitation Unit, Azienda Unità Sanitaria Locale IRCCS di Reggio Emilia, 42122 Reggio Emilia, Italy; silvia.sassi@ausl.re.it (S.S.); silvia.perazza@ausl.re.it (S.P.); silvia.sghedoni@ausl.re.it (S.S.); giulia.borelli@ausl.re.it (G.B.); adriano.ferrari@unimore.it (A.F.); 2 PhD Program in Clinical and Experimental Medicine, Department of Biomedical, Metabolic and Neural Sciences, University of Modena and Reggio Emilia, 41121 Modena, Italy; 3 Neurodevelopmental Unit, Fondazione IRCCS Istituto Neurologico Carlo Besta, 20133 Milan, Italy; emanuela.pagliano@istituto-besta.it (E.P.); maria.foscan@istituto-besta.it (M.F.); marta.vigano@istituto-besta.it (M.V.); 4 Functional Rehabilitation Unit, IRCCS E. Medea, Associazione La Nostra Famiglia, 23842 Bosisio Parini, Italy; cristina.maghini@lanostrafamiglia.it; 5 Physical Medicine and Rehabilitation Unit, S. Maria delle Croci Hospital, Azienda Unità Sanitaria Locale Romagna, 48121 Ravenna, Italy; mariafrancesca.siani@auslromagna.it; 6 Developmental Neuroscience Clinical Department, IRCCS Fondazione Stella Maris, 56128 Pisa, Italy; giada.sgherri@fsm.unipi.it (G.S.); ariannavaleria.bai@fsm.unipi.it (A.V.B.); 7 Physical Medicine and Rehabilitation Unit, IRCCS Istituto Ortopedico Rizzoli, 40136 Bologna, Italy; giuseppinamariagrazia.farella@ior.it

**Keywords:** physical therapy modalities, exercise, orthotic devices, patient participation, learning, play and playthings, physical and rehabilitation medicine, health services

## Abstract

Background: In 2020, a multiprofessional panel was set up in collaboration with the Italian FightTheStroke Foundation family association to produce evidence-based recommendations for the management and neuromotor rehabilitation of persons with cerebral palsy aged 2–18 years to implement in clinical practice in Italy. Methods: The recommendations of these care pathways were developed according to the American Academy for Cerebral Palsy and Developmental Medicine guidelines for Care Pathways Development and the Grading of Recommendations Assessment Development and Evaluation working group for adoption, adaptation, or de novo development of recommendations from high-quality guidelines (GRADE-ADOLOPMENT). Results: Four strong positive recommendations were developed regarding comprehensive management, and twenty-four addressed neuromotor treatment. Conclusions: A holistic, individualized approach was affirmed in terms of both multidimensional patient profile and interdisciplinary management in a network with the school where children and adolescents are integrated. It was defined that all motor rehabilitation approaches must be individually tailored considering age and developmentally appropriate activities as interventions and goals, in light of the reference curves addressing prognosis for Gross Motor Function and Manual Ability Classification Systems. Intervention must be structured with adaptations of the task and/or of the context (objects and environment) based on the analysis of the child’s skills to support motivation and avoid frustration.

## 1. Introduction

Cerebral palsy (CP) describes a group of permanent disorders of the development of movement and posture, causing activity limitation, which is attributed to non-progressive disturbances that occur in the developing fetal or infant brain. The motor disorders of cerebral palsy are often accompanied by disturbances of sensation, perception, cognition, communication, and behavior, by epilepsy, and by secondary musculoskeletal problems [1]. It is the most common motor disability in childhood, affecting 2–2.5 per 1000 live births [2].

In recent years, evidence regarding the management of patients with cerebral palsy has increased. Nonetheless, it is not always implemented in clinical practice, and differences are observed among service providers that can partially be attributed to their organization and their resources. Evidence-based care pathways are therefore recommended to orient clinical practice. In 2016, Italian healthcare providers agreed on and published recommendations concerning rehabilitation in CP, mainly focusing on general management [3]. In 2020, an interdisciplinary, multiprofessional panel (including physiatrists, pediatric neuropsychiatrists, physiotherapists, and neuro-psychomotor therapists representing their respective Italian scientific societies) was set up in collaboration with the FightTheStroke Foundation families’ association to update previous recommendations. Based on the fact that “the disorder of the development of movement and posture, causing activity limitation,” is the core of cerebral palsy, the panel agreed to focus on this aspect. Following the definition by the American Academy for Cerebral Palsy and Developmental Disability (AACPDM), care pathways are clinical recommendations based on the best evidence to manage and treat specific health conditions [4]. In line with the shift from the medical model of disability to the International Classification of Functioning (ICF) [5] perspective, the health condition was defined at the level of “activity limitation” rather than at “impairment”. The ICF interactive model identifies three levels of human functioning: at the level of body or body part, the whole person, and the whole person in his/her complete environment. These levels, in turn, define three aspects of functioning: body functions and structures, activities, and participation. Interventions at the level of body functions and structures may not necessarily determine an improvement at the activity level. Therefore, the panel decided to focus on the “Mobility” domain (d410-469) of the “Activity and Participation” components rather than on “Functions” or “Structures” dealing with posture and movement. 

The NICE guidelines [6,7] focus on several comorbidities and the management of pain, postural management to prevent secondary deformity, strengthening, and spasticity treatment (“Structures” and “Functions” domains) but do not specifically address the “Mobility” domain. The Australian guidelines [8] and the study by Novak et al. [9] address the “Activity and Participation” components, though they appraised the evidence up to 2019. Therefore, updating the findings was necessary.

Furthermore, to approach children and adolescents with CP as a whole person, the panel felt the need to provide some general statements defining the essential elements of the management of these patients. While international guidelines [6,7,8] state the need for multiprofessional management due to the presence of several comorbidities, a comprehensive vision is lacking, which leads to the risk of “breaking” the child down into pieces rather than approaching her/him as a whole person. Previous Italian recommendations [3] more comprehensively dealt with this topic, but they need to be updated. 

Therefore, a systematic review (SR) of the literature was conducted regarding the rehabilitation of children and adolescents with CP (detailed information about this SR has already been published [10]). A multidisciplinary panel then discussed the Evidence to Decision (EtD) framework and developed evidence-based recommendations suitable for the Italian context.

The aims of the present study were to report on the evidence-based development process and to present final recommendations regarding the management and motor rehabilitation of children and adolescents with CP, outlining the distinctive features of the adaptation to our national context.

## 2. Materials and Methods

The present study was conducted in accordance with the principles set forth in the Helsinki Declaration (1967). The approval of the local Ethics Committee was not necessary because no patient was directly involved. 

The Care Pathway panel included several healthcare providers as representatives of the following scientific societies: SIMFER (Italian Society of Physical and Rehabilitation Medicine), SINPIA (Italian Society of Paediatric Neuropsychiatry), SIRN (Italian Society of Neurologic Rehabilitation), AIFI_GIS (Italian Association of Physiotherapists_Paediatric Group), AITINE (Italian Association of Paediatric Neuro-Psychomotor Therapists), and ANUPI TNPEE (National Association of Paediatric Neuro-Psychomotor Therapists). These healthcare providers disclosed any existing conflicts of interest. One representative of the FightTheStroke Foundation family association participated. Discussion of the scope of the care pathway and subsequent activities were conducted through a few in-person and mostly online meetings due to COVID-19 pandemic restrictions. The care pathways apply to persons with CP aged 2–18 years and are dedicated to all types of healthcare providers involved in the management of persons with CP, as well as the patients, their caregivers, and their representatives as stakeholders.

The AACPDM guidelines for Care Pathways Development [4] and the Grading of Recommendations Assessment Development and Evaluation Evidence to Decision frameworks (EtD) [11] were followed to develop the recommendations of this care pathway. The PICO (Patients, Intervention, Control, Outcome) framework was used to structure the scope. 

After a panel discussion, two “critical” and two “important but not critical” topics were identified. The non-critical issues were the therapeutic relationship and the treatment contract between the rehabilitation professionals and the family. The present study, however, focused on the two “critical” questions:

1. What are the fundamentals of a comprehensive management of children and adolescents with CP?
2. Which rehabilitation approaches should be considered to improve gross motor or manual performance in children and adolescents with CP?

Regarding the PICO, the population considered were children and adolescents with CP aged 2–18 years. A separate care pathway was dedicated to the early treatment of children under age 2. 

The first query was necessarily broad to provide introductory statements defining a comprehensive way of caring for a child or adolescent with CP as a whole person. The second query focused on the “Mobility” domain at the “Activity” level [5] as the outcome. In particular, the panel was interested in interventions addressing activities connected with gross motor performance, corresponding to the “changing and maintaining body position” (d410–d429) and “walking and moving” (d450–d469) sections of ICF [5] and upper limb performance corresponding to the “carrying, moving and handling objects” (d430–d449) domains. These activities are strongly inter-related: posture and trunk alignment influence manual activities and the commonly used Gross Motor Function Measure includes items of postural control, walking, and transporting. Furthermore, many studies have examined mixed rehabilitation approaches to improve only one type of activity, while others have tested individual interventions (i.e., Hand-Arm Bimanual Intensive Therapy Including Lower Extremities, HABIT-ILE) to ameliorate two or all of these activities. Therefore, a single PICO was constructed that included any intervention addressing all the activities of the “Mobility” domain as possible outcomes so as to be more inclusive and thus avoid missing any information. Surgical and pharmacological approaches were excluded. No or any other treatment was accepted as the “control” item. 

Multiple databases [10] were systematically searched to answer each question.

The search focused on Clinical practice guidelines (CPGs) relative to CP management and rehabilitation first. Whenever the evidence was missing or incomplete, systematic reviews (SRs) and primary studies were enquired. CPGs were assessed using the Appraisal of Guidelines Research and Evaluation (AGREE) 2 tool [12,13]. SRs were assessed using the Assessing the Methodological Quality of Systematic Reviews (AMSTAR) 2 tool [14]. Primary observational studies were assessed using the Joanna Briggs Institute (JBI) Critical Appraisal Checklist for Case Series [15]. Screening, selection, quality assessments, and data extraction were independently carried out by two evaluators on the panel, who resolved any disagreement through discussion [10]. Afterward, the panel held an open discussion following the GRADE Evidence to Decision (EtD) framework [11]. The EtD framework facilitates the adoption or adaptation of existing recommendations or the production of de novo recommendations, considering criteria such as health benefits, certainty of best available evidence, and feasibility. Available recommendations were adopted or adapted, taking into account the criteria proposed by GRADE-ADOLOPMENT [11]. Whenever necessary, de novo recommendations were produced. In case of lack of evidence, the panel experts’ opinions were considered. After discussion, a consensus agreement on recommendations was reached. The panel then submitted a draft of the recommendations to external healthcare providers and to stakeholders through the FightTheStroke Foundation family association for external peer review.

## 3. Results

Our SR included four CPGs [3,6,7,8] and three primary studies [16,17,18] regarding Query 1 and two CPGs [7,8] and 43 SRs [9,19,20,21,22,23,24,25,26,27,28,29,30,31,32,33,34,35,36,37,38,39,40,41,42,43,44,45,46,47,48,49,50,51,52,53,54,55,56,57,58,59,60] concerning Query 2. 

The synthesis of the evidence (quality assessment and extracted data) and EtD frameworks relative to Query 1 are presented in detail in Tables S1 and S2, respectively.

**Figure 1:**
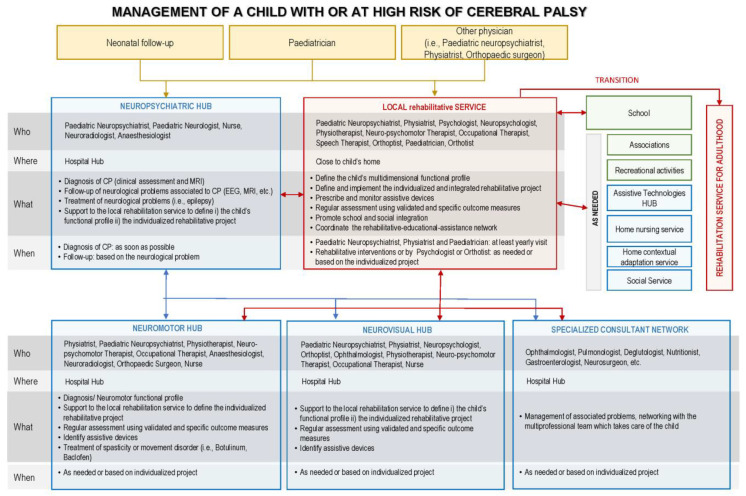
Flowchart representing the “Comprehensive management of children and adolescents with CP” care pathway with adaptation to the Italian context.

A flowchart was prepared to represent the “comprehensive management” care pathway adapted to the Italian context (Figure 1).

### 3.1. Four Strong Positive Recommendations Were Developed Regarding Comprehensive Management (Query 1):

1. Offer a management program oriented to specific goals that is individually tailored and that takes into consideration the following:
  - The needs and preferences of the young patient and his/her caregivers;
  - The multidimensional profile of functioning of the child (physical, mental, emotional, communicative, and relational);
  - Activities, as interventions and goals, that must be adequate to the age and the developmental stage;
  - Functional ability scales (Gross Motor Function Classification System, GMFCS; Manual Ability Classification System, MACS; Communication Function Classification System, CFCS; Visual Function Classification System, VFCS; Eating and Drinking Ability Classification System, EDACS), with reference curves addressing prognosis for GMFCS and MACS;
  - Evidence-based approaches;
  - Any implication for the patient and the caregivers and individual barriers;
  - Contextual barriers.
2. Regularly assess the child or adolescent by means of validated specific outcome measures to set realistic goals and verify their achievement; consequently, adapt the program;
3. Offer a transdisciplinary team approach, including all pediatric care professionals with expertise in CP management who may work in the same institution or as a network within the geographical area closest to the patient;
4. Ensure transition to adult services with expertise regarding cerebral palsy.

### 3.2. Query 2—“Motor Rehabilitation of Children and Adolescents with CP” Care Pathway

The synthesis of the evidence (quality assessment and extracted data) and EtD framework relative to Query 2 are presented in detail in Tables S3 and S4, respectively.

**Figure 2:**
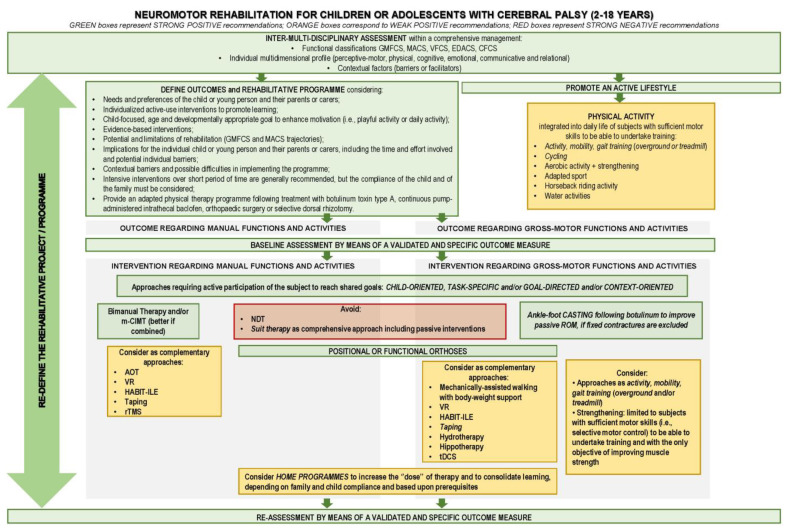
Flowchart representing the “Motor rehabilitation of children and adolescents with CP” care pathway with adaptation to the Italian context.

A flowchart was prepared to represent the “motor rehabilitation” care pathway with adaptation to the Italian context (Figure 2).

A total of 24 recommendations addressed neuromotor treatment (Query 2): 9 were “strong positive”, 11 were “conditional positive”, 2 were “strong negative”, and 2 were research recommendations. Evidence quality was from very low to high. The strong positive recommendations are reported below:

1. The motor rehabilitation interventions to improve gross motor or manual skills should consider the following issues:
  - Individualized and active;
  - Child-focused, age- and developmentally appropriate goals to enhance motivation;
  - The child’s multidimensional profile and environmental limitations;
  - Implications for the patient and his/her family;
  - The intervention should include individually tailored adaptations of the task and/or of the context (objects and environment) to help engagement and counteract frustration;
  - Repetitive practice of a task or part of it should be performed based on the child’s compliance;
  - Intensiveness increases effectiveness, but it is subordinated to the child’s compliance.
2. Consider bimanual interventions, such as performing functional tasks within enjoyable and playful activities, to improve bimanual skills in CP subjects. Bimanual interventions are intended to involve practicing a specific task or goal or parts of the task, focusing on the “activities” dimension rather than on “body and function”. Consider the need for minimum cognitive capacity as a requirement for bimanual training;
3. Consider modified Constraint Induced Movement Therapy (mCIMT) combined with bimanual therapy in unilateral CP to enhance manual skills. mCIMT might be applied up to two hours a day for a period of 2–10 weeks, performing functional tasks within enjoyable and playful activities. Consider reduced compliance and possible frustration, in particular with subjects with poorer function, as a limitation to applying mCIMT;
4. Consider the following factors in the selection of mCIMT or bimanual intensive interventions:
  - Preferences and peculiarities of the child or adolescent and his/her family features;
  - Expertise of the rehabilitation professionals involved;
  - Resources and service delivery models.
5. Provide an adapted physical therapy program following treatment with botulinum toxin type A, continuous pump-administered intrathecal baclofen, orthopedic surgery, or selective dorsal rhizotomy;
6. Consider task-specific, intensive, active rehabilitation to improve or recover after orthopedic surgery, as well as gross motor skills such as sitting, standing, balance, and gait. Equipment and orthoses may be used to assist in maintaining the person’s appropriate posture and movement;
7. As subjects with typical development, CP subjects with sufficient motor capacity should reduce sedentary behavior and perform light-intensity activities throughout the day as fitness training (i.e., gross motor activity training, cycling, overground or treadmill walking, modified sports). Nonetheless, the benefits, in terms of gross motor function and aerobic fitness, are short-lived and recede in case of suspension of training;
8. Consider upper and lower limbs:
  - Positional orthoses (i.e., leg or elbow orthoses, spinal braces) to maintain corrected anatomical alignment or range of motion of the joint and/or skin integrity;
  - Functional orthoses (i.e., ankle–foot, wrist, and thumb orthoses) to enable or improve function.
9. Consider ankle–foot casting following botulinum injection with the aim of improving dorsiflexion passive range of motion.

Conditional positive recommendations were provided for home-based programs to increase the training “dose”; Action Observation Therapy (AOT); HABIT-ILE; strengthening training; hydrotherapy; hippotherapy; treadmill training, with or without body weight support; virtual reality (VR) in terms of videogames for upper limb activities or balance, Non-Invasive Brain Stimulation (NIBS), in terms of either Transcranial Magnetic Stimulation (rTMS) or transcranial Direct Current Stimulation (tDCS), and taping as complementary approaches.

In line with previous CPGs, a strong negative recommendation was attributed to Neurodevelopmental Therapy (NDT) and to intensive suit therapy approaches.

Finally, research recommendations were produced to assess the effectiveness of Neuromuscular Electrical Stimulation (NMES), which includes Functional Electrical Stimulation (FES), in patients at GMFCS levels I–III after botulinum injections or orthopedic surgery and to assess the effectiveness of suits as “functional orthoses” to enable or improve function.

More details regarding the matching between instances from the evidence, the panel considerations, and decisions are reported in Supplementary Tables S3 and S4.

## 4. Discussion

The present care pathways (one per query) represent the result of a complex process of combining evidence-based findings with typical characteristics of the Italian cultural and scientific background in rehabilitation [61,62,63,64] and adapting them to the context. Some specific features distinguish the present work from international CPGs.

First of all, a holistic approach was affirmed in terms of both multidimensional patient profiles and interdisciplinary management. The international CPGs have already highlighted the need for a “multiple disciplinary” [8] approach, but the “multidimensional profile” is a characteristic of previous Italian recommendations [3], which we adopted in the present care pathways. In line with the interdisciplinary approach typical of the local context, the experts stated that any rehabilitative program should be tailored considering the individual as a whole person into his/her own social and environmental context (holistic and ecological approach). The motor, perceptive, cognitive, emotional, communicative, and relational functioning profiles should be taken into account as distinctive features. Moreover, the mutual influence between these areas should be considered together with the architecture of the compromised functions (activities/abilities). Furthermore, the individual goals should be appropriate to the age- and developmental stage. For this purpose, an interdisciplinary approach implemented by a multiprofessional team is necessary. In Italy, this approach involves a network of referral centers and local health services. The model involves longitudinal management by local services, which provide most rehabilitation interventions and assistive devices, and address the families to referral centers, which deal with diagnosis, associated neurological or psychiatric problems, visual impairment, neuromotor aspects (spasticity or dyskinesia treatment, functional surgery), feeding and nutritional problems, and so on. The organization of service and the integration between public and private healthcare facilities, however, vary at the regional level. One characteristic of Italy is its inclusive education: children and adolescents with any disability attend public school, and they receive individualized support to facilitate their learning and participation. This promotes inclusion of the person with a disability but primarily educates peers and society overall on diversity. The multiprofessional approach, therefore, should include school staff members who are involved in the network along with healthcare providers. The pediatric neuropsychiatrist should regularly meet with the school staff to help develop individualized curricula, and physiotherapists should conduct in-service training sessions of support staff and/or help to set up a context that facilitates the child’s learning.

One of the most sensitive issues for rehabilitation professionals is to answer parents’ questions relative to the prognosis of their children. The way professionals respond, in terms of either objective or emotional feedback, is crucial for the care relationship. Furthermore, figuring out a prognosis, at least in the short to middle term, is essential to hypothesize the expected results of treatment and to define the rehabilitative project. Rehabilitation may improve the level of functioning, activity, and participation of children and adolescents with CP, but based on the actual evidence, it cannot amend CP. Admitting the limitations of rehabilitation allows professionals to cautiously and pragmatically address the matter of prognosis. Therefore, the panel innovatively decided to introduce this topic into the care pathway. The Australian CPGs [8] recommend using functional motor ability classification scales to orient assessment and intervention (in particular, GMFCS [65] and MACS [66]). The panel decided to consider the developmental trajectories for gross motor and manual function reported in high-quality observational studies [16,17,18] to orient prognosis and identify the most critical periods for improvement. As Eliasson et al. reported, “the stabilizing of trajectories gives an important opportunity to shift the focus from capacity-related intervention to goal-directed training and participation interventions” [18]. This approach supports professionals in clarifying indications and limitations of rehabilitation and defining realistic individualized programs.

Another innovative feature is the inclusive and neuroscience-based perspective on “context” and “goal”. Evidence from the neurosciences underlines the role of contextual affordances mediated by canonic neurons [67,68] and of goals as an intrinsic part of the action that “resonates” in cerebral circuits involving mirror neurons [69,70,71]. Based on this, any action elicited by a child or adolescent within the rehabilitation intervention is influenced by contextual aspects, which must be considered as affordances. The therapeutic setting must therefore be carefully chosen. Furthermore, any action has an “intrinsic goal,” which identifies the action itself as significant for the child or adolescent [70]. This must be considered either in so-called “task-focused” interventions or in more strictly defined “goal-oriented” approaches. The panel therefore recommends considering both aspects as part of any intervention rather than opposing approaches, as often described in the literature.

Considering the role of affordances and perception in influencing individual behavior, the importance of taking into account any perception disturbances, first and foremost “tolerance”, becomes evident when planning any intervention [72] (i.e., selecting contextual stimuli based on the child’s tolerance is preferable to simply enriching the context). Therefore, the “implications for the individual child or young person” [7] encompass not only general compliance to any possible burden related to the intervention but, more specifically, the individual consequences of the exposure to all involved contextual elements.

Only bimanual therapy and mCIMT reached strong positive recommendations to improve bimanual performance in children with unilateral cerebral palsy. Both provide time-limited intensive practice of manual activities. Their effectiveness is comparable at a corresponding dose [7,8,9,27]. Therefore, the choice between the two must depend on the family’s preferences, the therapist’s expertise, and local resources. However, as these two approaches are not equivalent, some comments shall be provided. Bimanual therapy engages the patient in two-handed activities that induce specific bimanual actions and behaviors [73]. The interaction between the child, the object, and the task involves perceptual and cognitive processes underlying explicit learning [74]. mCIMT involves restraining the child’s less impaired upper limb from inducing repetitive use of the impaired limb in unimanual activities tailored to the individual skills. Minimal attention is required [75], and implicit learning is boosted [73]. Typically, activities requiring unimanual involvement are simpler than bimanual and would be performed by the dominant hand to minimize effort and optimize effectiveness [76]. Thus, mCIMT does not allow the subject to practice the assistive role of the more impaired hand in bimanual activities. Therefore, as Hoare et al. [73] suggested, the panel recommends considering mCIMT and bimanual therapy as complementary. mCIMT could be performed first to improve the unimanual capacity of the impaired hand. Then, bimanual therapy could be implemented to integrate this capacity in bimanual activities, in which the impaired hand assumes an assistive role, to boost bimanual skill development as daily activities require [73]. The Australian CPG [8] reports that mCIMT is more effective when applied to more compromised children. Conversely, the present care pathway, based on the experts’ opinion, outlines the risk of incurring frustration in more compromised patients due to difficulties in performing unimanual tasks with the impaired hand. Therefore, these children might not be compliant with mCIMT, and compliance is one priority to be considered.

Limited evidence was found regarding other interventions to improve manual performance. Therefore, only conditional positive recommendations are made. AOT is supported by a strong rationale [69,70,71,77,78], though future research is advisable to define the indications in terms of frequency, intensity, and suitable patients [9,29,30]. A severe motor and/or cognitive impairment might represent a contraindication based on experts’ opinions.

In accordance with international CPGs [7,8], an adapted physical therapy program is recommended to achieve gross motor skills (i.e., learn for the first time) or to re-achieve them after surgery. This statement was considered a strong positive by the present care pathway. Nonetheless, no clear indication was given about which type of adapted physical therapy should be provided. Several types of intervention are described in the literature to improve balance [41], sitting [22], mobility, and gait, although the evidence is low to very low. These approaches include gross motor activity training [38], mobility training [9], balance training [41], sit-to-stand or other activity training on the ground [34], and gait training [31,38,40]. Assistive devices, taping, and the aid of the therapist are included. In addition, the present care pathway states that an adapted physical therapy program should actively engage the child, considering individually tailored and realistic tasks and goals appropriate to age and developmental stage in sight of the GMFCS trajectories [16]. In line with international CPGs, the panel included resistance training among the recommended approaches for the sole purpose of increasing muscle strength [32,33,37,39], as there is no evidence of its effectiveness at the activity and participation level [39]. The panel recommends integrating it with activity-based training on coordination and endurance. As conditional recommendations, either overground or treadmill walking was considered to improve gait [9,39,43]. No specific indication was expressed regarding the parameters of weight relief devices because no evidence is available on this topic.

In line with international CPGs, a strong positive recommendation is expressed to implement physical activity into daily life to maintain mobility, which tends to get worse in adults with CP. Fitness training includes adapted sports [9,38] and strength, aerobic (overground or treadmill walking [8,9,39] and cycling [35]) or mixed-type training. Two limitations are outlined: the training is limited to children having sufficient motor skills to be able to participate, and the benefits of the fitness training are not maintained when it stops.

Only positive conditional statements are expressed for the other types of interventions due to limited evidence. Immersive VR and VR games [9,20,41,48,49,50,51,52] applied to a platform or a treadmill may engage the child in gross motor exercises, with the advantage of being able to measure and reproduce the characteristics of the exercise. Nonetheless, their feasibility depends on the technology resources available to health service providers. Hydrotherapy is considered a complementary intervention [8] that may be combined with other task-oriented approaches to improve gross motor performance [9,19] after orthopedic surgery or within fitness activities. Based on experts’ opinions, the panel outlines possible limitations: open wounds, the patient’s compliance, and contextual barriers.

The use of FES and NMES for children with cerebral palsy is controversial [8,9,24,31,54], and limited data are available on adverse effects and compliance. Therefore, the present care pathway advises future research to assess its effectiveness in ambulatory patients, in particular following botulinum injections or orthopedic surgery.

In line with Australian CPG [8], a strong positive recommendation is expressed for orthoses (either functional or positional) [8,58] and ankle–foot casting following botulinum injection [8,59]. The latter is contraindicated in case of advanced contractures or bony changes occurring at a joint.

### Limitations

The present care pathway provides a list of evidence-based interventions to improve gross motor or manual performance, with a few statements, mostly based on experts’ opinions, regarding the limitations or cautions of some approaches. More detailed indications are needed to identify who is eligible to undergo certain treatments, i.e., age, psychological and cognitive profile, type of CP, and so on. Nonetheless, the available evidence was too limited to allow us to define more specific indications. Further research studies on these topics are needed.

Another limitation of the care pathways developed in this study is that the entire process was long due to the COVID-19 pandemic [79], which monopolized rehabilitation services and limited in-person meetings. Therefore, an update will soon be necessary.

## 5. Conclusions

The traditional Italian multidimensional approach to children and adolescents with CP has evolved in tandem with neuroscience findings. The latter has explained and reinforced many of the issues that have emerged in rehabilitation practice. Neuroscience and rehabilitation proceed in partnership to further help each other interpret and modify human behavior. The present care pathways represent an attempt to synthetize the evidence from rehabilitation studies with neuroscience concepts and the multidimensional approach typical of the Italian context.

## Data Availability

Further detailed information relative to the included studies can be found in the supplementary material or are available at https://doi.org/10.3389/fneur.2023.1171224.

## Supplementary Materials

The following supporting information can be downloaded at https://www.mdpi.com/article/10.3390/children11070852/s1, Table S1: Synthesis of quality and contents of the evidence, panel considerations, and conclusions relative to Query 1, “What are the general principles to provide a comprehensive management of children and adolescents with CP?”; Table S2: Evidence to Decision framework relative to Query 1, “What are the general principles to provide a comprehensive management of children and adolescents with CP?”; Table S3: Synthesis of quality and contents of the evidence, panel considerations and conclusions relative to Query 2, “What are the most effective motor rehabilitation approaches to improve gross motor or upper limb performance in children and adolescents with CP?”; Table S4: Evidence to Decision framework relative to Query 2, “What are the most effective motor rehabilitation approaches to improve gross motor or upper limb performance in children and adolescents with CP?”.
