# Supplementary material for: Care Pathways in Rehabilitation for Children and Adolescents with Cerebral Palsy: Distinctiveness of the Adaptation to the Italian Context
Source: Children (Basel). 2024 Jul 13;11(7):852. doi: 10.3390/children11070852 (PMC11275177; doi:10.3390/children11070852)
Supplement: Supplementary file 1 [file children-11-00852-s001.zip › Table S1.pdf]

Table S1. Synthesis of quality and contents of the evidence, panel considerations and conclusions relative to Query 1 “What are the general principles to provide a comprehensive management of children and adolescents with CP?”

Reference	Publication Type	Quality assessment	Recommendations / Authors conclusions	Evidence to decision considerations	Panel conclusions
Spasticity in under 19s: management National Institute for Health and Care Excellence (NICE guidelines, 2012-2016; 2020)	CPG	AGREE II: high	1.1.5 Offer a management program that is:<br><br>developed and implemented in partnership with the child or young person and their parents or carers, **individualized**, **goal focused.**<br><br>1.1.6 When formulating a management program **take into account its possible impact on the individual child or young person and their family.**<br><br>1.1.8 Identify and agree with children and young people and their parents or carers assessments and goals that:<br><br>- are **age and developmentally appropriate**;<br><br>- **focus on** the following domains of the World Health Organization's International Classification of Functioning, Disability and Health**: body functions, body structures, activities and participation, environmental factors**.<br><br>1.2.2 Offer a physical therapy (physiotherapy and/or occupational therapy) program **tailored to the child or young person's individual needs and aimed at specific goals, such as: enhancing skill development, function and ability to participate in everyday activities; preventing consequences such as pain or contractures.**<br><br>1.2.4 When formulating a physical therapy program for children and young people **take into account**:<br><br>- **the views** of the child or young person and their parents or carers	The panel **adopts** all the issues, just reworks the statements to formulate exhaustive recommendations with the aim of including all the issues of the examined CPGs, prioritized according to the quality assessment.	1. Offer a management programme aimed at specific goals (e.g., enhancing skill development, function, and ability to participate in everyday activities; preventing secondary disorders such as pain, contractures and deformities) that is individually tailored and that takes into consideration:<br><br>- the needs and preferences of the child or adolescent and his/her parents or caregivers<br><br>- the multidimensional profile of the child (holistic approach), including physical, mental, emotional, communication and relational features<br><br>- age and developmentally appropriate activities as interventions and goals<br><br>- functional ability scales (GMFCS, MACS,

			- **the likelihood** of achieving the treatment goals<br><br>- **possible difficulties in implementing the program**<br><br>- **implications for the individual child or young person and their parents or carers, including the time and effort involved and potential individual barriers.**<br><br>1.2.5 When deciding who should deliver physical therapy, take into account:<br><br>- whether the child or young person and their parents or carers are able to deliver the specific therapy<br><br>- what training the child or young person, or their parents or carers might need<br><br>- the wishes of the child or young person and their parents or carers.<br><br>1.2.17 **Reassess** the physical therapy program at regular intervals to ensure that: the goals are being achieved; the program remains appropriate to the child or young person's needs".		CFCS, VFCS, EDACS), with reference curves addressing prognosis for GMFCS and MACS*<br><br>- evidence-based interventions<br><br>- implications (including emotional implications) for the individual child or adolescent and his/her parents or caregivers, including the time and effort involved and potential individual barriers<br><br>- contextual barriers and possible difficulties in implementing the programme<br><br>**STRONG +**
NICE guidelines: Managing cerebral palsy in under 25s 2021 http://pathways.nice.org.uk/pathways/cerebral-palsy	CPG	AGREE II: high	As a minimum standard of care, **ensure that the young person has access to adults' services both locally and regionally that include healthcare professionals with an understanding of managing cerebral palsy.**		2. Provide baseline and regular assessment of the child's or adolescent's functioning using validated ~~and~~ specific tools to ensure realistic goal setting, provide a baseline for therapy and verify whether the goals are being achieved and/or the programme remains appropriate to the child's or adolescent's needs.<br><br>**STRONG +**
Management Of Cerebral Palsy in Children: A Guide for Allied Health Professionals (NSW Ministry of Health guidelines, 2018)	CPG	AGREE II: high	The guideline reflects what is currently regarded as a safe and appropriate approach to the management of children with cerebral palsy. However, as in any clinical situation there may be factors which cannot be covered by a single set of guidelines. This document should be used **as a guide**, rather than as a complete authoritative statement of procedures to be followed in respect of each individual presentation. **It does not replace the need for the application of clinical judgement to each individual presentation.** As in any clinical situation and due to the heterogeneous nature of cerebral palsy, there are factors that cannot be covered by a single guide. **Clinicians and clients need to develop individual treatment plans that are tailored to the specific needs and circumstances of the client.** This guideline		3. Offer a multidisciplinary, interdisciplinary, or transdisciplinary team approach, including all paediatric care professionals

			should be read in conjunction with other relevant guidelines, position papers, codes of conduct, and policies and procedures, at professional, organizational and Local Health District levels.<br><br>**A multiple disciplinary team approach (whether in a format of multidisciplinary, interdisciplinary or transdisciplinary**), is considered best practice when working with children with complex needs. Research evidence supporting the effectiveness of a multiple disciplinary team approach is however, limited and shows conflicting results. Although a multiple disciplinary team approach may not be feasible in all settings, due to geographical, financial, organizational and time restraints, **where possible, it is recommended that all disciplines involved with the child work together in a family-centered model** to meet the needs and goals of the child and their family. Where clinicians may not work directly with, or have access to, other disciplines, they are encouraged to seek multidisciplinary **support from other disciplines within their organization or geographical area, or from tertiary institutions or specialist services to facilitate the provision of a holistic service.** Care must be taken to ensure that all professionals involved in a child's care are included and this may include **childcare professionals and/or teachers**. Particular attention should be focused on times of **transition** with early forward planning being essential for positive outcomes.<br><br>The **functional motor ability classification scales* should be used to guide assessment and intervention** with all children diagnosed with cerebral palsy to facilitate communication and goal setting. **Further assessment should occur to ensure realistic goal setting, provide a baseline for therapy and for evaluation of therapy programs**."		with expertise in CP management (paediatrician, neuropsychiatrist, physiatrist, physiotherapist, neuro-psychomotor therapists, occupational therapist, speech therapist, psychologist, orthopaedic surgeon, nurse, orthotist, etc.) who may work within the same organization or as a network within the geographical area closest to the child or adolescent, or at tertiary institutions or specialist services, and educational professionals, to facilitate the provision of a holistic service.<br><br>**STRONG +**<br><br>4. Ensure that the young person has access to adult services both locally and regionally that include healthcare providers with an understanding of how to manage cerebral palsy.<br><br>**STRONG +**
SIMFER-SINPIA Intersociety Commission. Recommendations for the rehabilitation of children with cerebral palsy (Eur J Phys Rehabil Med, 2016)	CPG	AGREE II: low	Rehabilitation is a complex process aimed at **promoting the best possible participation and quality of life for the child and for the family.** Through direct and indirect actions, it focuses on the individual in **all his dimensions**, physical, mental, emotional, communicative and relational (**holistic approach**), and it involves the child's family, social and environmental context (ecological approach).<br>The justified fields of intervention are determined on the basis of the data relating to the **patient's profile**, and are related to:<br>— the architecture of the main functions (activities/abilities) on which to intervene for therapeutic purposes (the focus of the re-education plan);<br>—the types of these main functions, which can fall within the following areas: autonomic control, personal autonomy,		

			locomotion, manipulation and praxis, sensation/perception and gnosis, cognition, communication, relationships;<br>—the compatibility of the therapeutic targets with the activities/abilities and the levels of participation appropriate to the age group considered;<br>—the priority functional activities/abilities and the levels of participation that the child with CP should, **considering his specific age range, succeed in attaining; in other words, the developmental stages** (windows for intervention/critical periods). In this sense, the priority functional activities/abilities do not respect a predetermined hierarchical order (milestones), but change depending on the child's age group. For example, walking is an important goal between the ages of 0 and 2 years and between the ages of 3 and 5 years and, in certain situations, can continue to be so between 6 and 8 years of age, but after this time it ceases to be an important goal, except in exceptional, justified circumstances. Conversely, the achievement of adequate autonomy in the sitting position becomes very important in children who use a manual or electronic wheelchair, an aid that, moreover, can be proposed for patients as young as 3 to 5 years of age, if they have a negative prognosis for walking….<br>The assessment of the patient must take into account **not only the single functional area involved, but also its relationship with the other areas, so as to be able to define the overall level of development attained and the impact, on this, of the area in question**. In seeking to characterize the elements comprising the different functional areas, it is important to provide not just a mere description of the phenomenon (it is present / it is not present / it is partially present / it is emerging), but also to state whether and in what way the child implements adaptive, compensatory or additional strategies, not least because these can serve as a crucial guide for the proposed therapy.		
Rosenbaum 2002	Longitudinal cohort study	JBI: high	657 uni-bilateral CP, mixed types, age 1-13 ys at first assessment, follow-up 4 ys. Gross motor development curves were presented, describing average development predicted by the Gross Motor Classification System. **Higher ability levels reached their limit of development in longer period than lower ability levels, though all levels reached their developmental limit by the age of 7 ys.**	The Australian CPG recommends: "The functional motor ability classification scales* should be used to guide assessment and intervention". Recent evidence demonstrated the prognostic value of such classification scales for gross motor and manual function. The panel then included this evidence to	* Considering gross motor and bimanual performance and all ability levels, CP children reach their developmental limit by the age of 7 ys, later improvements are possible for individual skills in a goal-oriented approach.
Klevberg 2018	Prospective longitudinal study	JBI: high	60 unilateral, 42 bilateral CP, mixed types, MACS I-III, age at first assessment (mo): unilat. 25.0 (range 18–58), bilat. 35.0 (range 18–59); mean follow-up 4.5 months (range 5–51 mo). Although the AHA and BoHA are different tests, and the data are not directly comparable, children with **bilateral CP seem to change their performance over time to a smaller extent than those with unilateral CP**. Furthermore, children with **bilateral CP seem to reach their developmental limits around 30 months of age,**		

			**regardless of MACS level**. More research is needed to further explore these findings, and particularly for children with bilateral CP, more knowledge is warranted to understand their specific challenges and to explore interventions that may potentially enhance their development. Nevertheless, our results call for **early hand function interventions to facilitate the long-term development of bimanual performance**, with particular attention to the grasping or holding ability and the asymmetric hand use at early age.	implement the recommendation 1:<br>– reference prognostic curves for gross motor performance in mixed CP GMFCS I-V: higher ability levels reached their limit of development in longer period than lower ability levels, though all levels reached their developmental limit by the age of 7 ys;<br>– reference trajectories for bimanual performance in bilateral mixed CP MACS I-III: they reach their developmental limits around 30 months of age; then very early interventions are recommended;<br>– reference trajectories for bimanual performance in unilateral spastic CP MACS I-III: they reach their developmental limit by the age of 7 years, later the level is stable, but improvement can be reached relative to individual skills by means of a goal-directed training.	
Eliasson 2022	Prospective longitudinal study	JBI: high	171 unilateral spastic CP, age range 18 mo-18 ys, mean follow-up 8 ys (range 1–17). Previous knowledge that **the AHA score at 18 months together with the MACS levels is predictive of future development** was confirmed in this larger study. Children classified as having higher ability (MACS level I) had both a higher rate and limit of development and a shorter period of development than those having a lower ability (MACS level II). Children functioning in MACS level III had the lowest limit, and development occurred during the longest time. **The stable performance lasted throughout adolescence for participants in all MACS levels from approximately 7 years.** This demonstrates that, if the children have learned to use both hands in a meaningful way, they continue to use them when growing up. On an individual level, large variation in development is seen; therefore, regular follow-ups for children in all MACS levels in the clinic are important. Furthermore, the stabilizing of trajectories gives an important opportunity **to shift the focus from capacity-related intervention to goal-directed training and participation interventions.** There is positive evidence that children at any age and functional level can learn new skills.		
