# Supplementary material for: Care Pathways in Rehabilitation for Children and Adolescents with Cerebral Palsy: Distinctiveness of the Adaptation to the Italian Context
Source: Children (Basel). 2024 Jul 13;11(7):852. doi: 10.3390/children11070852 (PMC11275177; doi:10.3390/children11070852)
Supplement: Supplementary file 1 [file children-11-00852-s001.zip › Table S2.pdf]

Table S2. Evidence to Decision framework relative to Query 1 “What are the general principles to provide a comprehensive management of children and adolescents with CP?”

Recommendation 1

Offer a management programme aimed at specific goals (e.g., enhancing skill development, function, and ability to participate in everyday activities; preventing secondary disorders such as pain, contractures and deformities) that is individually tailored and that takes into consideration:

- the needs and preferences of the child or adolescent and his/her parents or caregivers
- the multidimensional profile of the child (holistic approach), including physical, mental, emotional, communication and relational features
- age and developmentally appropriate activities as interventions and goals
- functional ability scales (GMFCS, MACS, CFCS, VFCS, EDACS), with reference curves addressing prognosis for GMFCS and MACS
- evidence-based interventions
- implications (including emotional implications) for the individual child or adolescent and his/her parents or caregivers, including the time and effort involved and potential individual barriers
- contextual barriers and possible difficulties in implementing the programme

Type of recommendation		Adopted x<br>Adapted □<br>De novo □	
FACTOR	DECISION		EXPLANATION
Balance of benefits	Benefits outweigh disadvantages x<br>Benefits and disadvantages are balanced □<br>Disadvantages outweigh benefits □		A comprehensive and individually tailored management is essential to address individual needs and orient the investment of the resources.
Quality of the evidence	High x<br>Moderate □<br>Low □<br>Very low □		High quality guidelines confirmed by the expert opinions among the panel supported the recommendation.
Values and preferences	No significant variability x<br>Significant variability □		All agreed.
Resource required	Large costs □<br>Moderate x<br>Low □<br>Don’t know □		A better organised and oriented approach optimizes the costs of the management of patients with disability due to a chronic condition.
Acceptability	Yes x<br>No □<br>Don’t know □		The approach is in favour of individual needs and preferences of patients and their families.
Feasibility	Yes x<br>No □<br>Don’t know □		The approach considers contextual barriers and possible difficulties in implementing the program.
Overall strength and direction of the recommendation	Strong x<br>Conditional □	Positive x<br>Negative □	

Recommendation 2

Provide baseline and regular assessment of the child’s or adolescent’s functioning using validated specific tools to ensure realistic goal setting, provide a baseline for therapy and verify whether the goals are being achieved and/or the programme remains appropriate to the child’s or adolescent’s needs.

Type of recommendation		Adopted x<br>Adapted □<br>De novo □	
FACTOR	DECISION		EXPLANATION

<table>
<tr><td>Balance of benefits</td><td colspan="2">Benefits outweigh disadvantages x
Benefits and disadvantages are balanced □
Disadvantages outweigh benefits □</td><td>Regular assessment allows orienting following investment of patient and family commitment and services' resources.</td></tr>
<tr><td>Quality of the evidence</td><td colspan="2">High x
Moderate □
Low □
Very low □</td><td>High quality guidelines confirmed by the expert opinions among the panel supported the recommendation.</td></tr>
<tr><td>Values and preferences</td><td colspan="2">No significant variability x
Significant variability □</td><td>All agreed.</td></tr>
<tr><td>Resource required</td><td colspan="2">Large costs □
Moderate x
Low □
Don't know □</td><td>Regular assessment allows orienting following investment of services' resources.</td></tr>
<tr><td>Acceptability</td><td colspan="2">Yes x
No □
Don't know □</td><td>Assessments are usually well tolerated and currently executed in most clinical context.</td></tr>
<tr><td>Feasibility</td><td colspan="2">Yes x
No □
Don't know □</td><td>Feasible with appropriate information and training.</td></tr>
<tr><td>Overall strength and direction of the recommendation</td><td>Strong x
Conditional □</td><td>Positive x
Negative □</td><td></td></tr>
</table>

<table>
<tr><td colspan="4">Recommendation 3
Offer a multidisciplinary, interdisciplinary, or transdisciplinary team approach, including all paediatric care professionals with expertise in CP management (paediatrician, neuropsychiatrist, physiatrist, physiotherapist, neuro-psychomotor therapists, occupational therapist, speech therapist, psychologist, orthopaedic surgeon, nurse, orthotist, etc.) who may work within the same organization or as a network within the geographical area closest to the child or adolescent, or at tertiary institutions or specialist services, and educational professionals, to facilitate the provision of a holistic service.</td></tr>
<tr><td colspan="2">Type of recommendation</td><td colspan="2">Adopted x
Adapted □
De novo □</td></tr>
<tr><td>FACTOR</td><td colspan="2">DECISION</td><td>EXPLANATION</td></tr>
<tr><td>Balance of benefits</td><td colspan="2">Benefits outweigh disadvantages x
Benefits and disadvantages are balanced □
Disadvantages outweigh benefits □</td><td>Creating and maintaining the network might be onerous and time consuming for the professionals, but enable a comprehensive and individually tailored management.</td></tr>
<tr><td>Quality of the evidence</td><td colspan="2">High x
Moderate □
Low □
Very low □</td><td>High quality guidelines confirmed by the expert opinions among the panel supported the recommendation.</td></tr>
<tr><td>Values and preferences</td><td colspan="2">No significant variability x
Significant variability □</td><td>All agreed.</td></tr>
<tr><td>Resource required</td><td colspan="2">Large costs □
Moderate x
Low □
Don't know □</td><td>The costs involved to create and maintain the network or multiprofessional teams counterbalance losses due to inappropriate or redundant interventions.</td></tr>
<tr><td>Acceptability</td><td colspan="2">Yes x
No □
Don't know □</td><td>No disadvantages for the patients.</td></tr>
</table>

Feasibility	Yes x<br>No □<br>Don’t know □		Feasible orienting resources to build multiprofessional teams or networks, instead of preserving local interests.
Overall strength and direction of the recommendation	Strong x<br>Conditional □	Positive x<br>Negative □	

**Recommendation 4**<br>Ensure that the young person has access to adult services both locally and regionally that include healthcare providers with an understanding of how to manage cerebral palsy.			
Type of recommendation		Adopted x<br>Adapted □<br>De novo □	
FACTOR	DECISION		EXPLANATION
Balance of benefits	Benefits outweigh disadvantages x<br>Benefits and disadvantages are balanced □<br>Disadvantages outweigh benefits □		Ensuring an appropriate transition to adult services reduces emerging problems in adulthood.
Quality of the evidence	High x<br>Moderate □<br>Low □<br>Very low □		High quality guidelines confirmed by the expert opinions among the panel supported the recommendation.
Values and preferences	No significant variability x<br>Significant variability □		All agreed.
Resource required	Large costs □<br>Moderate x<br>Low □<br>Don’t know □		The costs involved to ensure access to appropriate services for adult patients counterbalance losses due to inappropriate interventions or management of secondary deformities.
Acceptability	Yes x<br>No □<br>Don’t know □		No disadvantages for the patients.
Feasibility	Yes x<br>No □<br>Don’t know □		Feasible but an overall reorganization of services is needed.
Overall strength and direction of the recommendation	Strong x<br>Conditional □	Positive x<br>Negative □	
