# Supplementary material for: Care Pathways in Rehabilitation for Children and Adolescents with Cerebral Palsy: Distinctiveness of the Adaptation to the Italian Context
Source: Children (Basel). 2024 Jul 13;11(7):852. doi: 10.3390/children11070852 (PMC11275177; doi:10.3390/children11070852)
Supplement: Supplementary file 1 [file children-11-00852-s001.zip › Table S3.pdf]

Table S3. Synthesis of quality and contents of the evidence, panel considerations and conclusions relative to Query 2 "What are the most effective motor rehabilitation approaches to improve gross motor or upper limb performance in children and adolescents with CP?"

Interventions	Reference	Publication Type	Quality assessment	Recommendations / Authors conclusions	Evidence to decision considerations	Panel conclusions
CHILD-FOCUSED THERAPY (GOAL/ TASK ORIENTED TRAINING) versus CONTEXT-FOCUSED THERAPY	Spasticity in under 19s: management National Institute for Health and Care Excellence (NICE guidelines, 2012-2016; 2020)	CPG	AGREE II: high	1.1.5 Offer a management program that is: developed and implemented in partnership with the child or young person and their parents or carers, individualized, goal focused.<br>1.1.6 When formulating a management program take into account its possible impact on the individual child or young person and their family.<br>1.1.8 Identify and agree with children and young people and their parents or carers assessments and goals that:<br>- are age and developmentally appropriate;<br>- focus on the following domains of the World Health Organisation's International Classification of Functioning, Disability and Health: body functions, body structures, activities and participation, environmental factors.<br>1.2.2 Offer a physical therapy (physiotherapy and/or occupational therapy) program tailored to the child or young person's individual needs and aimed at specific goals, such as: enhancing skill development, function and ability to participate in everyday activities; preventing consequences such as pain or contractures.<br>1.2.4 When formulating a physical therapy program for children and young people take into account:<br>- the views of the child or young person and their parents or carers;<br>- the likelihood of achieving the treatment goals;<br>- possible difficulties in implementing the program;<br>- implications for the individual child or young person and their parents or carers, including the time and effort involved and potential individual barriers.	The panel **adopts** issues relative to:<br>- Individualized and goal-focused;<br>- Impact on the child and family;<br>- Age and developmentally appropriate;<br>- Tailored on needs and specific goals;<br>- Consider implications for the individual child and family, individual barriers;<br>- Task-focused active-use therapy;<br>- Intensive over a short time.	**1.** The motor rehabilitation approaches to improve gross motor or manual skills should consider the following issues:<br>- individualized active-use interventions;<br>- child-focused, age and developmentally appropriate goal to enhance motivation (i.e., playful activity or daily activity);<br>- the task should be analysed considering the child's skills, as well as environmental limitations;<br>- consider not only motor skills but the child's multidimensional profile;<br>- consider the impact of the intervention on the child and the family;<br>- intervention might be structured with adaptations of the task and/or of the context (objects and environment), based on the analysis of the

				1.2.11 Consider task-focused active-use therapy such as constraint-induced movement therapy (temporary restraint of an unaffected arm to encourage use of the other arm) followed by bimanual therapy (unrestrained use of both arms) to enhance manual skills.<br>1.2.12 When undertaking task-focused active-use therapy consider an intensive program over a short time period (for example, 4–8 weeks).		child's skills, to support motivation and avoid frustration;<br><br>- intervention should involve repetitive practice of the task or part of it, without incurring burnout in the child;<br><br>- intensive interventions over short periods of time have generally been found to be more effective, but the compliance of the child and of the family must be considered.<br><br>**STRONG +**
	Management Of Cerebral Palsy In Children: A Guide For Allied Health Professionals (NSW Ministry of Health guidelines, 2018)	CPG	AGREE II: high	PAG.90 There are four main stages involved in goal directed therapy. The first is the formation of an age and developmentally appropriate goal. Goals should always be child focused to increase motivation. Assessment to identify the goal limiting factor(s) is a crucial next step. The task should then be analysed, considering the child's skills as well as environmental limitations. Intervention should be structured and involve repetitive practice, appropriate adaptations to the task or the environment and outcomes evaluated using validated tools.<br>PAG.91 Context focused therapy consists of changing the task or the environment (but not the underlying body structure and function of the child) to promote successful task performance. In context focused therapy activities are identified which a child likes or needs to do but has difficulty doing. The focus is then on changing the activity to make it easier to do by reducing restricting factors in the environment or the task. A child will practice activities within context, and individualised strategies are determined for each child and family member.<br>Therapy that focuses on changing the activity and the environment is considered as effective in improving functioning as therapy focusing on changing the child.	The panel **adopts** issues relative to:<br><br>- goal directed therapy, as child-focused, considering an age and developmentally appropriate goal, with intervention structured involving repetitive practice, with appropriate adaptations to the task or the environment;<br><br>- context focused therapy as effective as child-focused.	
	Jackman et al. 2020	SR	AMSTAR 2: very low	74 RCT or quasi-RCT Active interventions were subcoded as "goal-directed", "functional or part-task", or "non-functional". CP subjects or high risk of CP, age 0-18 years. Outcome measures: AHA, COPM.<br><br>To improve individual goals, children need to practice goals for more than 14–25 hr, combining face-to-face therapy with home practice. To improve general upper limb function (based on evidence in the unilateral population), children need to practice for more than	The panel discusses the risk of indicating such definite doses of interventions, due to the need for individualised approaches based on the characteristics of the subjects. A limitation of the included studies was the short or absent follow-up.<br><br>It is considered an interesting issue that interventions that set functional goals	

				30–40 hr. Interventions that set functional goals and involve actual practice of those goals lead to goal achievement at a lower dose than general upper limb motor training.<br><br>Dose of practice is one important factor in considering the ideal upper limb intervention for children with CP. Children are likely to achieve individual goals, if they have set their own goals and have practised those goals for more than 14 to 25 hr. To improve motor ability, a higher dose of practice is needed, likely 30 to 40 hr of practice.<br><br>If the purpose of the intervention is to achieve individual goals, goal-directed interventions, in which goals are practised, rather than focussing on underlying skills, are more effective.<br><br>For goal-directed or functional training, where the outcome was measured on the AHA, logistic regression showed that children under 8 years of age were 2 times more likely to succeed. On the COPM results were similar regardless of age, although children over 8 years were 1.46 times more likely to succeed.	and involve the actual practice of those goals lead to goal achievement with a lower dose than general upper limb motor training.<br><br>The panel raises the consideration that younger children might be more responsive to functional or part-task training than older subjects, who may still improve individual goals with a goal-directed training (as confirmed by Eliasson 2022).	
	SIMFER-SINPIA Intersociety Commission. Recommendations for the rehabilitation of children with cerebral palsy (Eur J Phys Rehabil Med, 2016)	CPG	AGREE II: low	The justified fields of intervention are determined on the basis of the data relating to the patient’s profile, and are related to:<br><br>—the architecture of the main functions (activities/abilities) on which to intervene for therapeutic purposes (the focus of the re-education plan);<br><br>—the types of these main functions, which can fall within the following areas: autonomic control, personal autonomy, locomotion, manipulation and praxis, sensation/perception and gnosis, cognition, communication, relationships;<br><br>—the compatibility of the therapeutic targets with the activities/abilities and the levels of participation appropriate to the age group considered;		

				—the priority functional activities/abilities and the levels of participation that the child with CP should, considering his specific age range, succeed in attaining; in other words the developmental stages (windows for intervention/critical periods).<br><br>The assessment of the patient must take into account not only the single functional area involved, but also its relationship with the other areas, so as to be able to define the overall level of development attained and the impact, on this, of the area in question.		
	Novak et al. 2019	SR	AMSTAR 2: moderate	1 SR, GMFCS I-III 4-18 yrs. Relative to GOAL ORIENTED TRAINING the authors confirm data from previous SR (Novak et al.,2013: 5 RCT): "There are now numerous RCTs in the upper and lower limb, plus in early intervention, cognitive orientation to occupational performance, HABIT-ILE and in the home program evidence based, consistently indicating that goal directed training improves goal achievement of functional tasks involving motor performance. We upgraded the recommendations for use from weak positive to strong positive" to improve gross motor, hand function and self-care.<br>2 RCT, GMFCS I-III 4-18 yrs. Relative to TASK ORIENTED TRAINING the authors included into the analysis only 2 small RCTs that "conferred improved gross motor skills compared to control non-task-based therapy. More research is needed".<br>3 RCT, GMFCS I-IV, 11 mo–4 yrs. Relative to CONTEXT FOCUSED THERAPY the authors reported "No between group differences for context focussed v child focussed v usual care" to improve selfcare in GMFCS levels I-V.<br><br>"Since context focussed is equally effective to child focussed, it is recommended that both approaches are used simultaneously, or the family selects the one that suits their preferences. Status update since 2013: The field has come to understand more about how to specifically train improvements in self-care skills through task specific training, goal-directed training and cognitive orientation to occupational performance. With this added data, the effect size of	The panel considers that distinguishing context versus child-oriented therapies and task-oriented versus goal-oriented training addresses the need to categorize the interventions for research studies focusing on the predominant aspect.<br><br>Nonetheless, in clinical practice an overlap of these issues is often observed, even in research studies the distinction is not always so clear. Then the panel decides to retain the issues as components of the rehabilitation approach in a more inclusive and general perspective.	

				context focussed therapy is now less clear given that is compared to child-focussed therapy &/or usual care, which is an umbrella term for many different therapies with varying effect sizes. We have therefore downgraded the quality from high to moderate and recommendations for use from strong positive to weak positive."		
	Inamdar et al. 2021	SR and meta-analysis	AMSTAR 2: high	12 RCT. Uni-bilateral CP, GMFCS I-V, 18 mo-puberty. Components of impairment remediation combined with functional balance training should be explored to improve sitting in children diagnosed with CP.<br>Task-specific, intensive, and child-initiated intervention components show promise for improving sitting in young infants at risk for CP. Given the benefits of early achievement of sitting, strong evidence-based research is needed.		
	Hsu et al. 2019	SR and meta-regression	AMSTAR 2: low	13 RCT. GMFCS I-III, 1-17 yrs. Meta-regression analysis revealed that the improvement in GMFM scores was positively associated with the number of daily training hours and program duration.		
	Das et al. 2019	SR	AMSTAR 2: very low	34 SR. 0-18 yrs, mostly hemiplegic CP. Intensive activity-based, goal-directed interventions are more effective. The ability of manual stretching to increase range of motion and reduce spasticity is limited.		

BIMANUAL TRAINING / HABIT	Spasticity in under 19s: management National Institute for Health and Care Excellence (NICE guidelines, 2012-2016; 2020)	CPG	AGREE II: high	1.2.11 Consider task-focused active-use therapy such as **constraint-induced movement therapy** (temporary restraint of an unaffected arm to encourage use of the other arm) followed by bimanual therapy (unrestrained use of both arms) to enhance manual skills.	The panel **adopts** recommendations relative to combining CIMT and bimanual task 1.2.211	**2.** Consider bimanual interventions, performing functional tasks within enjoyable and playful activities, to improve bimanual skills in CP subjects. Bimanual interventions are intended as involving practising a specific task or goal, or parts of the task, focusing on the "activities" dimension rather than on "body and function". Consider the need for minimum cognitive skills to respond to cues, as a requirement for bimanual training.
	Management Of Cerebral Palsy In Children: A Guide For Allied Health	CPG	AGREE II: high	PAG. 88 **Bimanual training** provides an increased opportunity to practice bilateral activities with the goal of leading to an improved use of both hands during activity. Bimanual training involves practising the specific task or goal, or parts of the task, rather than	The panel **adopts** the recommendation relative to **bimanual training** as involving practising the specific task or goal, or parts of the task, and focusing on the "activities" dimension rather than	

	Professionals (NSW Ministry of Health guidelines, 2018)			focussing on the underlying body structure and function deficits. There is reliable evidence to support the use of bimanual therapy, with outcomes of bimanual therapy being equal to that of CIMT when the same amount of therapy is provided. Best candidates for bimanual training are typically older than 12 months, have spontaneous use of affected hand, selective motor control, have basic skills such as grasp and hold and have the cognitive skills to respond to cues.	"body and function" (pag.88). The panel agrees on the need of minimal cognitive skills to respond to cues.	**STRONG +**
	Alahmari et al. 2020	SR and Meta-analysis	AMSTAR 2: moderate	4 RCT, CP hemiplegia, duration of intervention 60-90 h over a period of 2-4 weeks. Meta-analysis on the efficacy (measured by means of JTHFT) of HABIT versus CIMT or structured and unstructured bimanual therapy, HABIT showed a trivial effect compared to the other interventions, with an effect size of 0.06. Both groups performed functional tasks improving hand function within enjoyable and playful activities.	Confirm the effectiveness of bimanual intensive interventions and CIMT.<br><br>The panel agrees with the importance of performing functional tasks to improve hand function within enjoyable and playful activities.	
	Ouyang et al. 2020	SR	AMSTAR 2: low	11 RCT, 1 quasi-RCT, 1 retrospective, 2 longitudinal studies. Individualised training, group-based training, or both, mostly in daily camp, for hemiplegic CP age 3-18 years. Mixed outcome measures: AHA, JTHFT, QUEST, ABILHAND-Kids, BBT, COPM, PEDI.<br>HABIT in the form of 6 h a day for 3 consecutive weeks (totaling 90 h) led to the improvement of bimanual ability, unilateral dexterity, self-care function, and functional goals after the intervention and that the improvements were mostly maintained during the follow-up period (duration not specified). HABIT with different dosages or added training components showed evidence for improving self-care function and functional goals, there was little impact on upper extremity function.	Confirm the effectiveness of bimanual intensive interventions and CIMT.	
	Novak et al. 2019	SR	AMSTAR 2: moderate	3 RCT, CP hemiplegia 2-10 yrs. Duration intervention 90 h. "CIMT was equally effective for improving bimanual performance and unimanual capacity as dose matched OT or HABIT (bimanual training). All systematic reviews that compare CIMT with Bimanual Intensive Training concluded that both approaches led to similar improvements in upper limb function. Since CIMT is equally effective to<br>bimanual, it recommended that both approaches are used, or the family selects the one that suits their preferences."	Confirm that bimanual training and CIMT are equally effective at the same dose.<br>Confirm to use both or to rely on the family preferences.	

CIMT	Spasticity in under 19s: management National Institute for Health and Care Excellence (NICE guidelines, 2012-2016; 2020)	CPG	AGREE II: high	1.2.11 Consider task-focused active-use therapy such as **constraint-induced movement therapy** (temporary restraint of an unaffected arm to encourage use of the other arm) followed by bimanual therapy (unrestrained use of both arms) to enhance manual skills.<br><br>1.2.12 When undertaking task-focused active-use therapy consider an intensive program over a short time period (for example, 4–8 weeks).	The panel **adopts** recommendations relative to combining CIMT and bimanual task 1.2.211 and **adapt** recommendation 1.2.12, considering that there are discrepancies between the CPGs, relative to the duration and the intensity of mCIMT.	**3.** Consider mCIMT combined with bimanual therapy in unilateral CP, to enhance manual skills. mCIMT might be applied up to two hours a day for a period of 2-10 weeks, performing functional tasks within enjoyable and playful activities. Consider reduced compliance and possible frustration, in particular with subjects with poorer function, as a limitation to applying mCIMT.<br><br>**STRONG +**
	Management Of Cerebral Palsy In Children: A Guide For Allied Health Professionals (NSW Ministry of Health guidelines, 2018)	CPG	AGREE II: high	PAG. 87 **CIMT** with children with cerebral palsy has involved the use of slings, mitts, splints and casts applied for most of the waking day, for a set period of weeks. Concerns regarding the intensity of the intervention has led to a modified model where the constraint is applied for up to two hours a day but for a longer overall duration. Outcomes of modified Constraint Induced Movement Therapy (**mCIMT**) have been shown to be just as effective as CIMT. The evidence indicates that mCIMT is more effective than usual care and the model of treatment appears to be age dependent. Under the age of 4 years shorter periods of daily practice at home and/or preschool over an 8 to 10 weeks period is effective while in children over 4 years of age intensive 2 to 3 weeks camps or group-based intervention appears more effective. Higher intensity does not always result in better outcomes and CIMT is not age dependent, although children with poorer hand function do tend to make greater improvements.	The panel **adopts** the recommendation relative to mCIMT, such as applying the constraint up to 2 hours a day for longer period than CIMT (pag.87), and **adapt** the recommendation concerning:<br>- duration/intensity;<br>- "Children with poorer function tend to make greater improvement".<br>The panel outlines the risk that "children with poorer function" might experience frustration while treated with CIMT.<br>The panel recommends considering compliance and frustration of the child as a limitation to applying mCIMT.	**4.** Consider the following factors in the selection of mCIMT or bimanual intensive interventions:<br>- child/adolescent and family characteristics and preferences<br>- therapist's expertise<br>- costs of implementing the intervention;<br>- funding and service delivery models;<br>- resource availability.<br><br>**STRONG +**
	Hoare et al. 2018	SR and meta-analysis	AMSTAR 2: high	36 RCT Unilateral CP, mean age 5.96 years (3 m- 19.8 yrs). The most common constraint devices were a mitt/glove or a sling (11 studies each). Frequency 2-7 days/week, duration of intervention sessions 0.5-8/hours per day, for a period of 1-10 weeks). Mixed outcome measures: AHA, QUEST, MUUL, BBT, ABILHAND-Kids. CIMT appears no more effective than another upper-limb therapy that is carried out intensively (most comparisons with intensive bimanual interventions that were therapist-led and more clearly defined). CIMT did not appear to impact body structure and function outcomes, such as grip strength, muscle stiffness and spasticity, and had no consistent effect on quality of life. Although there was minimal research on participation outcomes, it is	The panel agrees on the instance of considering the child and family characteristics and preferences, therapist expertise, costs of implementing the intervention, funding and service delivery models and resource availability, in the selection of mCIMT or bimanual intensive interventions.	

				hypothesised that CIMT and bimanual interventions may not have a direct effect on children's participation. Two key ingredients across all models of CIMT are maintained: 1) restraint of the well-functioning upper limb (irrespective of device/type); 2) intensive, structured training (irrespective of type). Clinicians, therefore, should view CIMT as a relatively short-term intervention that is provided for a defined period, and carefully evaluate outcomes before and aJer implementation using valid and reliable measures. CIMT appears to be a safe intervention for children with unilateral CP. Families should feel confident that, on average, active engagement in a well-defined, intensive program of CIMT or bimanual therapy can lead to improvements in bimanual performance and unimanual capacity. This review was not able to identify the characteristics of children who could be advised to participate in one or the other of CIMT or bimanual interventions. In the meantime, clinicians should consider the specific goals for individual children and families and choose the most developmentally appropriate, family-centred, and convenient of these approaches (Hoare 2017). Factors, in addition to child and family characteristics and preferences, which may impact on intervention selection include therapist expertise, costs of implementing the intervention, funding and service delivery models, and resource availability.		
	Novak et al. 2019	SR	AMSTAR 2: moderate	1 SR (Hoare et al., 2018), CP hemiplegia, 3 mo – 19 yrs; 1 SR (Chiu et al., 2016), CP hemiplegia 2-10 yrs. The authors strongly recommended CIMT to improve bimanual performance and unimanual capacity in hemiplegic CP. They also recommended CIMT to improve activity and participation: "CIMT conferred better activity and participation gains than no therapy, with large effect sizes. CIMT was equally effective to dose matched OT for producing activity and participation gains. Authors proposed the mechanism for improvements relates to intensity of practice rather than the type of intervention, consistent with the conclusions of previous reviews".	Data support evidence in favour of mCIMT to improve unimanual function, bimanual performance, activity and participation in hemiplegic CP, compared to no treatment. Confirm the importance of intensive practice, as the key issue, without more specifications.	
	Das et al. 2019	SR	AMSTAR 2: very low	34 SR. 0-18 yrs, mostly hemiplegic CP. There is positive support for the use of CIMT to improve the upper-extremity functioning.		

HOME PROGRAMMES	Spasticity in under 19s: management National Institute for Health and Care Excellence (NICE guidelines, 2012-2016; 2020)	CPG	AGREE II: high	1.2.5 When deciding who should deliver physical therapy, take into account:<br>-whether the child or young person and their parents or carers are able to deliver the specific therapy<br>-what training the child or young person or their parents or carers might need<br>-the wishes of the child or young person and their parents or carers.	The panel adapts the recommendation based on the large variability of studies' characteristics in recent SR.	**5.** Home programmes might be considered to increase the "dose" of therapy, to improve the performance of functional activities, depending on family and child compliance and based upon the following five step model:<br>1. establish collaborative relationships between parents and therapist;<br>2. set mutually agreed upon family and child goals;<br>3. select therapeutic activities focused on achieving family and child goals, that are supported by best available evidence;<br>4. support implementation of home programmes through parent education, home visits and programmes update to sustain motivation;<br>5. evaluate outcomes.<br><br>**WEAK +**
	Management Of Cerebral Palsy In Children: A Guide For Allied Health Professionals (NSW Ministry of Health guidelines, 2018)	CPG	AGREE II: high	PAG.90 There is strong evidence to support the effectiveness of home programs aimed at improving the performance of functional activities when based upon the following five step model.<br>1. Establish collaborative relationships between parents and therapist<br>2. Set mutually agreed upon family and child goals<br>3. Select therapeutic activities that focus on achieving family and child goals that are supported by best available evidence<br>4. Support implementation of home program through parent education, home visits and program updates to sustain motivation<br>5. Evaluate outcomes.<br>There is currently insufficient evidence to support the use of home programs aimed at improving participation. The use of appropriate outcome measures for evaluation is recommended.	The panel adapts the recommendation based on the large variability of studies' characteristics in recent SR	
	Beckers et al. 2020	SR	AMSTAR 2: low	30 effectiveness study (26 RCT, 4 single subject studies), CP uni/bilateral, GMFCS I-V, 4 mo-19 yrs. Conclusions about the effectiveness of home programs cannot be made due to the large variability in the study, patient and intervention characteristics, comparators, and outcome measures used in the included studies. Even within the same treatment approach, frequency and duration of the interventions varied. As training intensity is an important predictor for treatment success, improvement in arm-hand function and performance can therefore not be solely attributed to the intervention approach. …<br>The question which motor learning approach in the specific context of parent-delivered programs is best suitable, remains, therefore, unanswered…. Coaching of parents is a key element of home-based programs.		

	Novak et al. 2019	SR	AMSTAR 2: moderate	2 RCT, GMFCS I-V, 4-13 yrs. "Home programs conferred improved function compared to control no therapy…they are an effective way to increase the dose of therapy."		
AOT	Abdelhaleem et al. 2021	SR and meta-analysis	AMSTAR 2: high	12 RCT, uni/bilateral CP, 5-15 yrs. Duration of intervention …no evidence of benefit had been found to draw a firm conclusion regarding the effectiveness of AOT in the rehabilitation of children with CP due to limitations in methodological quality and variations between studies	The evidences are still limited and future research is needed to verify effectiveness, optimal frequency and intensity of AOT programs, characteristics of children that better fit AOT approach.	**6.** AOT might be considered to improve bimanual performance in CP children, with particular attention to the severity of motor impairment and cognitive status as possible limitations.<br><br>**WEAK +**
	Alamer et al. 2020	SR	AMSTAR 2: low	9 RCT, CP hemiplegia, GMFCS I-IV, 3-12 yrs. Taken together, these findings suggest action observation therapy was found to be a better rehabilitative intervention for upper limb rehabilitation to improve physical function and structure, activities, and participation of children with hemiplegic CP than simple motor training. However, considerable attention must be paid when we use AOT for CP children, due to the severity of motor impairment, and cognitive status; further studies are needed to determine the optimal frequency, intensity, and time of AOT on these particular study subjects		
	Novak et al. 2019	SR	AMSTAR 2: moderate	2 RCT, unilateral spastic CP, ambulatory 5-15 yrs. Duration of intervention 1 hr/day for 15 days, for 3 wks. Upper limb action observation training conferred better bimanual performance compared to watching videos, but with a small effect size		
HABIT-ILE	Novak et al. 2019	SR	AMSTAR 2: moderate	2 RCT, uni/bilateral CP, GMFCS I-IV, 6-16 yrs. Duration of intervention: 90 hrs in camp format. Low evidence of improved motor function in both lower and upper limbs compared to usual care.	Low evidence	**7.** HABIT-ILE might be considered to improve upper and lower limb motor function in GMFCS I-IV subject age 6-16 yrs.<br><br>**WEAK +**
ADAPTED PHYSICAL THERAPY / PHYSICAL ACTIVITY	Spasticity in under 19s: management National Institute for Health and Care Excellence (NICE guidelines, 2012-2016; 2020)	CPG	AGREE II: high	1.2.13 Consider **muscle-strengthening therapy** where the assessment indicates that muscle weakness is contributing to loss of function or postural difficulties.<br>1.2.14 Direct muscle-strengthening therapy towards specific goals using progressive repetitive exercises performed against resistance.<br>1.2.15 Following treatment with botulinum toxin type A, continuous pump-administered intrathecal baclofen, orthopaedic surgery or selective dorsal rhizotomy, provide an **adapted physical therapy program** as an essential component of management.	The panel adapt recommendations 1.2.13-14:<br>the panel evidences that subjects with low selective-motor control are expected to be unable to achieve **muscle-strengthening**, with the risk of eliciting pathological cocontraction (due to spasticity or dystonia). The panel then recommends to consider **muscle-strengthening therapy** limited to subjects and body districts that express selective-motor control.	**8.** Provide an adapted physical therapy programme following treatment with botulinum toxin type A, continuous pump-administered intrathecal baclofen, orthopaedic surgery or selective dorsal rhizotomy.<br><br>**STRONG +**

				1.2.16 Ensure that children and young people and their parents or carers understand that an adapted physical therapy program will be an essential component of management following treatment with botulinum toxin type A, continuous pump-administered intrathecal baclofen, orthopaedic surgery or selective dorsal rhizotomy	The panel adopts recommendations 1.2.15-16: provide an adapted physical therapy program following treatment with botulinum toxin type A, continuous pump-administered intrathecal baclofen, orthopaedic surgery or selective dorsal rhizotomy.	**9.** Consider task-specific, intensive, and child-initiated intervention to achieve or re-achieve gross motor skills such as sitting, standing, balance and gait after an intervention (i.e., orthopaedic surgery). Equipment and orthoses may be utilised to assist in maintaining the person's appropriate posture and movement.<br><br>**STRONG +**
	Management Of Cerebral Palsy In Children: A Guide For Allied Health Professionals (NSW Ministry of Health guidelines, 2018)	CPG	AGREE II: high	PAG 79**. Strengthening programs and resistance training** is an accepted intervention for children with cerebral palsy, in particular lower limb strengthening. Various systematic reviews of the literature into strengthening indicate that the<br>effectiveness of strength training, particularly in the lower limbs, is still disputed but that clinical practice continues despite the lack of published evidence. The literature also indicates there are no adverse increases in spasticity arising from strength training programs. There is limited evidence regarding the effects of strengthening programs on activity and participation level outcomes. A critically appraised topic investigating strength training in the upper limbs of children with cerebral palsy found limited evidence to suggest that strengthening programs may increase upper limb strength in<br>children with cerebral palsy. Strength training/ progressive resistance exercise is based on three principles:<br>• To perform a small number of repetitions until fatigue<br>• Allow sufficient rest between exercises for recovery<br>• To increase the resistance as the ability to generate force increases.<br>Strengthening programs applied to children with cerebral palsy can be based on the guidelines published by The American Academy of Paediatrics and the National Strength and Conditioning Association (NSCA). Due to its intensity and the need for the muscles to rest and recover, it is not meant to be performed frequently and for long durations. Strength training needs to be combined with other activity-based programs such as treadmill training or cycling where you can look at other aspects of function such as endurance or coordination.	The panel adapts recommendation about **strengthening**:<br>● for lower limbs only, considering the limited evidence of effectiveness for upper limbs;<br>● evidence of effectiveness in muscle strength, not in gross motor function.	**10.** As for subjects with typical development, reducing sedentary behaviour and encouraging light intensity activities throughout the day as **fitness training** (i.e., gross motor activity training, cycling, overground or treadmill walking, modified sports) should be integrated into daily life of CP subjects with sufficient motor skills to be able to undertake training. Consider that fitness training may provide short-term benefits, in terms of gross-motor function and aerobic fitness, but these are not maintained when training stops.<br><br>**STRONG +**

				PAG. 90 **Fitness training**. Exercise can be defined as "planned structured activities involving repeated movement of skeletal muscles that result in energy expenditure to improve or maintain levels of physical fitness". Physical fitness is "a set of attributes that people have or achieve that relates to the ability to perform physical activity". The major components of health-related fitness are cardiorespiratory fitness and muscle strength. The primary and secondary impairments of cerebral palsy affect both cardiorespiratory fitness and muscle strength contributing to reduced physical fitness. Current evidence suggests that fitness training to improve aerobic fitness provides short-term benefits for clients with sufficient motor skills to be able to undertake training and any increase in capacity following training is not maintained when training stops. There is currently insufficient evidence to support the use of fitness training to improve function and participation however the research would suggest that aerobic fitness does not translate into either activity or participation gains. Frequency and intensity of interventions vary across the literature and generally focus on structured moderate to vigorous exercise. Attention is shifting with a growing recognition of the importance of reducing sedentary behaviour and encouraging light intensity activities throughout the day. It is recommended that fitness training to improve aerobic fitness, muscle strength and the general health of children with cerebral palsy should be integrated into the child's daily life on an ongoing basis.<br><br>PAG.88 **Gait training** is the process of first learning or re-learning how to walk after an intervention such as orthopaedic surgery and can be used as a therapy intervention for persons with cerebral palsy.<br>It can be achieved in a number of ways, but repetition of the actual motions/gait pattern performed during walking is the most important factor. Depending on the severity of the person's impairment, one or more physiotherapists may be present to assist in maintaining the person's appropriate posture and moving their lower limbs to assist in facilitating the prerequisites of a normal gait pattern. Parallel bars may be used to help with gait training, especially in the	The panel adopts recommendation about **fitness training**:<br>● to improve aerobic fitness;<br>● short-term benefits which are not maintained when training stops;<br>● for clients with sufficient motor skills to be able to undertake training;<br>● should be integrated into the child's daily life.<br><br>The panel adapt recommendation on gait training, including approaches to improve gross motor skills and achieve balance (standing) and sitting based on SRs (Clutterbuck 2019, Elnahhas 2019, Albuquerque de Araujo 2019, Inamdar 2021).	

				early stages of rehabilitation as the bars provide support for the child, and the clinician facilitates the desired movement. Other equipment, such as high support and low support assistive mobility devices are also utilised.		
	Corsi et al. 2021	SR	AMSTAR 2: low	13 RCT for gait or strength training. Uni-bilateral CP, GMFCS I-III, 7-18 yrs. Vibratory platform, gait training, electrical stimulation, and transcranial stimulation were effective to improve spatiotemporal gait parameters, especially velocity in children with cerebral palsy. Isolated strength training was not effective to improve gait parameters in Cerebral Palsy.	Do not modify CPGs.	
	Liang et al. 2021	SR and meta-analysis	AMSTAR 2: low	27 RCT. Uni-bilateral, GMFCS I-III, mean age 1.8-16 yrs. Exercise interventions (resistance or aerobic or mixed training) may have beneficial effects on gait speed and muscle strength, but no significant effect on gross motor function in children with CP.	Do not modify CPGs.	
	Merino-Andres et al. 2021 (Cho et al. 2020)	SR and meta-analysis	AMSTAR 2: high	27 RCT (same studies included in Ryan et al. 2017, except Cho et al. 2020, which is included only in meta-analysis regarding "Balance" as outcome: 25 bilateral CP). Uni-bilateral CP, GMFCS I-IV, 3-22 yrs. Significant standardised mean differences in favour of strength training program compared to other physical therapy technique(s) or untreated control group(s) for muscle strength at the knee flexors, at the knee extensor, at the plantarflexors, maximum resistance, balance, gait speed, GMFM (global, D and E dimension) and spasticity.	Do not modify CPGs.	
	Bania et al. 2019	SR and meta-analysis	AMSTAR 2: very low	9 RCT. GMFCS I-III, 2 (most were over 6)-18 yrs. Activity training on the ground (whole -body self-initiated activities such as sitting, turning, sit-to-stand, walking, stepping, stair climbing, or other similar activities people use to transfer independently or with a handheld support in home or outdoor settings) compared to no treatment or usual treatment (NDT or strengthening): no statistically significant difference. Low evidence.	Do not modify CPGs.	
	Armstrong et al. 2019	SR and meta-analysis	AMSTAR 2: high	5 RCT, 1 quasi-RCT, 1 comparison trial, 1 pre–post study with a control group, 1 single-group study with a control period. GMFCS I-V, uni-bilateral CP-mean age 10.4 yrs (SD 2.3). Cycling can improve aerobic fitness, muscle strength, balance and gross motor function in children with CP; however, optimal training doses are yet to be determined. There was	Do not modify CPGs.	

				insufficient data to determine whether functional improvements can be retained. Conclusions were limited by small sample sizes, inconsistent outcome measures and a lack of follow-up testing.		
	Lopez et al. 2019	SR	AMSTAR 2: very low	1 case study, 10 clinical trials (3 RCT), 3 pilot studies. Children and adults. The potential for dance and Rhythmic auditory stimulation (RAS) to have positive impacts on body functions, emotional expression, social participation, and attitudinal change are indicated areas for consideration in future research.	Do not modify CPGs.	
	Das et al. 2019	SR	AMSTAR 2: very low	34 SR. 0-18 yrs, mostly hemiplegic CP. Intensive activity-based, goal-directed interventions are more effective. The ability of manual stretching to increase range of motion and reduce spasticity is limited.<br>Gait speed can be improved most effectively by gait training	Do not modify CPGs.	
	Collado-Garrido et al. 2019	SR	AMSTAR 2: high	12 RCT, 3 non RCT. Uni-bilateral CP, GMFCS I-V, 4-18 yrs. …statistically significant positive effect on motor function in favor of the use of resistance therapy in weakened musculature in children with CP. Resistance therapy would not only increase the strength of the musculature of children with CP, but this increased strength would also have an impact on the motor function. Nevertheless, it is possible that the between-group effect may have been overestimated due to the existence of a publication bias. Necessary to review and modify the games and exercise used to perform the resistance therapy, so as guarantee adherence.	Do not modify CPGs.	
	Clutterbuck et al. 2019	SR	AMSTAR 2: very low	34 studies (17 RCT). CP mixed types, GMFCS I-IV (mostly I-III), 3-18 yrs. Active exercise interventions (Gross Motor Activity Training alone or with progressive resistance exercise plus additional physiotherapy, Physical Fitness Training, Modified Sport, Non-Immersive Virtual Reality) improve gross motor function of ambulant/semi-ambulant children with CP. Gross Motor Activity Training is the most common and effective intervention. Practice variability is essential to improve gross motor function. Participation was rarely measured and requires further research, particularly in interventions that embed real-world participation opportunities like Modified Sport.	This SR supports (low level evidence) active exercise interventions to improve gross motor function of ambulant/semi-ambulant children with CP.	
	Novak et al. 2019	SR	AMSTAR 2: moderate	**Fitness**. Moderate-based evidence (including Ryan 2017). Effective for children GMFCS I-II who can move fast enough to train aerobic fitness. … aerobic	Confirm that fitness (including cycling and treadmill), mobility training, modified sports, physical activity may	

				exercise (including cycling and treadmill) conferred improved gross motor function in the short and intermediate term, did not improve gait speed.<br>**Mobility training.** 6 SR. GMFCS I-IV, 3-21 yrs. Low to moderate-level evidence of improving gait speed and gross motor function. Eclectic group of interventions including Nintendo, wall climbing, sit to stand, circuit training of functional tasks, overground or treadmill walking.<br>**Modified sport.** Observational studies. CP mixed types, GMFCS I-III, 4-16 yrs. Very low-level evidence of improving gross motor skills, gait speed, aerobic fitness.<br>**Physical activity.** 4 SR. CP mixed types, GMFCS I-V, <25 yrs. Low-level evidence, conflicting results on improving gross motor function, gait and fitness.<br>**Strength training.** 4 SR. Mixed CP types, GMFCS I-III, 3.4-20 yrs. Strength Training conferred improved muscle strength and gait.	improve gross motor function; strength training improves muscle strength.	
	Ryan et al. 2017	SR and meta-analysis	AMSTAR 2: high	29 RCT (4 included adults; 8 compared aerobic exercise to usual care, 15 compared resistance training to either usual care or no treatment, 4 compared mixed training to usual care or no treatment, 2 compared aerobic exercise to resistance training) evaluated as low to very low-level evidence. CP mixed types, GMFCS I-V <19 yrs. Aerobic exercise may improve activity as indicated by motor function but does not appear to improve gait speed, walking endurance, participation or aerobic fitness among children with CP in the short or intermediate term. There is no research regarding the effect of aerobic exercise on participation or quality of life. Resistance training does not appear to improve motor function, gait speed or participation in the short or intermediate term, or quality of life in the short term, in children and adolescents with CP but may improve muscle strength. Mixed training does not improve motor function or gait speed but does improve participation in children and adolescents with CP in the short term. We found no difference between aerobic and resistance training on motor function but a difference in muscle strength in the short term. Although the evidence suggests that exercise might be safe for people with CP, only 16 trials (55%) included information on adverse events; these trials reported no serious adverse events. All the	Aerobic exercise may improve motor function; resistance training does not improve motor function but improves muscle strength, though supported by low to very low evidence.	**11.** Strengthening training for lower limbs is an accepted intervention for CP children with sufficient motor skills (i.e., selective motor control) to be able to undertake training, with the only objective of improving muscle strength. It requires a small number of repetitions until fatigue, rest periods, short duration, and low frequency. Strengthening training does not improve motor function.<br><br>**WEAK +**

				studies we found had small numbers of participants, meaning that we cannot be sure the results are accurate		
	Elnahhas et al. 2019	SR	AMSTAR 2: low	7 RCT. Uni-bilateral spastic CP, GMFCS I-III, 5-14 yrs.<br>Moderate evidence that backward gait training improves mobility (gait) and some evidence that backward gait training improves balance and gross motor function.	Low evidence: backward gait improves gait.	
	Albuquerque de Araujo et al. 2019	SR and meta-analysis	AMSTAR 2: low	7 RCT. Uni-bilateral spastic CP, GMFCS I-II (incomplete data), 5-15 yrs. Very-low quality evidence suggests that balance-training interventions (i.e., activities that cause unpredicted perturbations, such as unstable or mobile surfaces, in multiple training settings) combined with other intervention enhances the effect of the other intervention alone on postural control at short-term.	Very-low level evidence: balance training interventions in multiple training settings contribute to improve postural control.	
	Inamdar et al. 2021	SR and meta-analysis	AMSTAR 2: high	12 RCT. Uni-bilateral CP, GMFCS I-V, 18 mo-puberty. Components of impairment remediation combined with functional balance training should be explored to improve sitting in children diagnosed with CP.<br>Task-specific, intensive, and child-initiated intervention components show promise for improving sitting in young infants at risk for CP. Given the benefits of early achievement of sitting, strong evidence-based research is needed.	Task-specific, intensive, and child-initiated intervention components show promise for improving sitting in young infants at risk for CP.	
	Yardımcı-Lokmanoglu et al. 2020	SR	AMSTAR 2: very low	3 small RCT. Spastic CP, GMFCS I-III, 5-15 yrs. Different approaches on proprioception (i.e., whole body vibration or integrated intensive proprioceptive and visuomotor training) combined with conventional physiotherapy (CPT), showed no superiority on motor performance, compared to CPT alone.	Do not modify CPGs.	
HYDROTH ERAPY	Management Of Cerebral Palsy In Children: A Guide For Allied Health Professionals (NSW Ministry of Health guidelines, 2018)	CPG	AGREE II: high	PAG. 96 The warmth and buoyancy of the water provides support which can aid pain relief, but also a different movement experience to that on land. The heat of the water may assist relaxation or help reduce spasms. Walking may not only be easier but possible without aides for some children and young adults with cerebral palsy. Fitness and endurance can be more easily challenged in a controlled way. Hydrotherapy is also an excellent recreational pursuit which can lead to improved swimming skills, and respiratory function. … reported benefits such as improved function for children with cerebral palsy, including better walking efficiency, improved strength, range of motion or	The panel adapt the recommendation: based on clinical experience the panel consider that hydrotherapy might help in rehabilitation programs following orthopedic surgery, in particular bone orthopedic surgery. Possible limitations: open wounds, child's compliance, context's barriers, and services' resources.	**12.** Consider hydrotherapy as a complementary approach in rehabilitation programmes, to recover gross motor function following orthopaedic surgery, to improve fitness and endurance. It must be differentiated from recreational motor activity into the water or adapted sport, which is advisable, whenever possible, to improve fitness. Possible limitations: open wounds,

				balance. However, furthermore robust studies were needed.		child's compliance, contextual barriers, and services' resources.<br><br>**WEAK +**
	Roostaei et al. 2017		AMSTAR 2: very low	11 studies (2 RCT). Uni-bilateral CP mixed types, GMFCS I-V, 3-21 yrs. Frequency 2-3/week, duration 6–16 weeks. Evidence on aquatic interventions for ambulatory children with CP is limited. Aquatic exercise is feasible and adverse effects are minimal; however, dosing parameters are unclear. Further research is needed to determine aquatic intervention effectiveness and exercise dosing across age categories and GMFCS levels. The aquatic setting, specifically type of pool, temperature of the water, and group or individualized instruction should be specified.	Limited evidence, but feasible and adverse effects are minimal.	
	Novak et al. 2019	SR	AMSTAR 2: moderate	Low-quality supporting evidence (including Roostaei 2017). Aquatic-based exercises improved vitals and gross motor function.	Low-level evidence for improvements in gross motor function.	
TREADMILL/MECHANICALLY ASSISTED WALKING	Management Of Cerebral Palsy In Children: A Guide For Allied Health Professionals (NSW Ministry of Health guidelines, 2018)	CPG	AGREE II: high	PAG.88 Treadmill training is an active approach to gait training in which the child practices the movement of walking on a treadmill rather than within the real-world environment. Treadmill training may include partial body-weight support, in which the child is placed in a harness that supports their weight, whilst a clinician manually guides the legs in a walking motion. Treadmill training, including those with partial body-weight support, are based on motor learning theories, in which the child carries out the activities of walking repetitively, with increasing speed and weight-bearing with the aim of this skill carrying over to walking within an everyday context. Combined results from four systematic reviews suggest that there is low quality evidence to support treadmill training to improve weight-bearing. It also found low quality evidence that treadmill training will improve functional walking although the practice of overground walking, rather than treadmill training may be more effective.	The panel adapts the recommendation based on the following studies, considering:<br>- treadmill training with or without body weight support, as **one** type of exercise that permits repetitions and aerobic training, then can improve gait endurance and aerobic fitness;<br>- evidence regarding effectiveness of body weight support treadmill compared to no walking.<br>Nonetheless, the panel outlines differences among services relative to organisation and resources, as possible contextual barriers.	**13.** Consider treadmill training, with or without body weight support, as one possible alternative approach to overground walking, to improve gait endurance, gait speed and gross motor function. Being just **one** type of gait training approach, consider resources and preferences of subjects and their families, and of service providers.<br><br>**WEAK +**<br><br>Future research is required to define need and parameters of body-weight support and motion guidance, for mechanically assisted walking devices.
	Chiu et al. 2020	SR and meta-analysis	AMSTAR 2: high	17 RCT. Uni-bilateral CP, GMFCS I-IV, 4-14 yrs. Duration of the intervention 4-12 weeks, intensity of training 15-40 minutes, frequency 2-5/week.<br>Compared with no walking, mechanically assisted walking training probably results in small increases in walking speed (with or without body weight support) and may improve gross motor function (with body weight support). Compared with the same dose of	Treadmill with body weight support may improve gross motor function compared to no walking, but no significant difference is observed compared to the same dose of overground walking	

				overground walking, mechanically assisted walking training with body weight support may result in little to no difference in walking speed and gross motor function, although two studies found that mechanically assisted walking training without body weight support is probably more effective than the same dose of overground walking training for walking speed and gross motor function. Not many studies reported adverse events, although those that did appeared to show no differences between groups. The results are largely not clinically significant, sample sizes are small, and risk of bias and intensity of intervention vary across studies, making it hard to draw robust conclusions.<br>Mechanically assisted walking training is a means to undertake high-intensity, repetitive, task-specific training and may be useful for children with poor concentration.		
	Han et al. 2020	SR and meta-analysis	AMSTAR 2: moderate	8 RCT. Uni-bilateral CP, GMFCS I-IV, mean age 4.5-16 yrs. These findings suggested that treadmill training on cerebral palsy was effective for gait endurance, gait speed and limb support time. No significant improvement in cadence and step length.	Moderate evidence in favour of treadmill, to improve gait endurance and speed.	
	Novak et al. 2019	SR	AMSTAR 2: moderate	3 SR. Uni-bilateral CP, GMFCS I-IV, 4-21 yrs. Treadmill training, with or without body weight support, conferred improved walking speed, endurance and gross motor function.	Moderate evidence in favour of treadmill, to improve gait endurance and speed.	

VR - VIDEOGAMES	Johansen et al. 2020	SR and Meta-analysis	AMSTAR 2: moderate	8 RCT, GMFCS I-V, 5-20 yrs. …the results highlight the potentials of videogames as a supplementary method of training of arm and hand function for persons with CP. Results should be interpreted with caution due to high risk of bias and low strength of evidence (positive: task oriented, motivating, intensive).	The evidence is limited: the panel outlines possible difficulties relative to costs and appeal with technology of rehabilitative services, professionals and/or families.	**14.** Consider virtual reality in terms of videogames to improve hand function in an individualized rehabilitation programme, as a complement to conventional therapies. Possible limitations: child's compliance, contextual barriers and resources.<br><br>**WEAK +**
	Plasschaert et al. 2019	SR	AMSTAR 2: low	2 studies. Bilateral CP. Very low-level evidence about improvement in upper limb function.		
	Rathinam et al. 2019	SR	AMSTAR 2: low	6 RCT, uni/bilateral CP, GMFCS I-V, 6-18 yrs. 4 studies reported some improvement in hand function, but only one had a low risk of bias. .... The existing reviews reported the improved motivational factor and high parental satisfaction, but our study did not examine this component. Our review is in agreement		

				with the other reviews that the available evidence from the existing studies was inconsistent and that VR cannot be reliably suggested to improve hand function until further studies have ascertained its therapeutic effect.		
	Novak et al. 2019	SR	AMSTAR 2: moderate	1 SR (19 RCT), CP 4-12 yrs. Duration of intervention 20-90 mins/ day, 1-7x wk, over 4- 20 wks. VR conferred better arm function than NDT or usual care, with large effect sizes… Findings suggest that games are to be used as a complement to conventional therapies and not as a substitute. VR may also induce neuroplasticity.		
VR-GROSS MOTOR/ BALANCE	Montoro-Cardenas et al. 2021	SR	AMSTAR 2: high	GMFCS I-IV, spastic uni/bilateral Nintendo WII Balance (NWT) can be considered an effective treatment for improving functional and dynamic balance in children with CP, especially when combined with CPT (Conventional Physical therapy) in 30-minute sessions with interventions lasting longer than 3 weeks. Accessible and low cost. Incorporates motivation and fun. On functional balance we found very low quality evidence with large effect of NWT compared with no intervention and moderate quality evidence for using NWT (plus conventional) CPT versus CPT in session of approximately 30 min and interventions lasting longer than 3 ws; for dynamic balance very low quality evidence for a medium effect for using NWT plus CT versus CPT was found.	The evidence is limited, and the panel underlines possible difficulties relative to costs and appeal with technology of rehabilitative services, professionals and/or families.	**15.** Consider virtual reality games (i.e., Nintendo WII Balance) as an additional treatment for improving functional and dynamic balance in children with CP at GMFCS level I-IV, combined with conventional physical therapy. Possible limitations: child's compliance, contextual barriers, and resources. **WEAK +**
	Wu et al. 2021	SR	AMSTAR 2: very low	11 RCT. Hemiplegic, diplegic, quadriplegic CP. GMFCS I-IV but incomplete data about GMFCS levels, age over 6 yrs. VR games played a positive role in the improvement of balance of children with CP, but these results should be viewed with caution owing to current methodological defects.		
	Ren et al. 2019	SR	AMSTAR 2: very low	7 RCT. Hemiplegic, diplegic, quadriplegic CP. GMFCS I-V, age over 6 yrs. Preliminary evidence shows that VRGs have positive effect on the improvement of gross motor skills of children with CP. Additionally, the single intervention time was 17–40		

				min, the intervention frequency should be over 5 times per week, the intervention cycle was over 12 weeks while the total intervention time should be more than 1000 min. VRGs essentially belongs to the functional training that based on the ecological theory and dynamical systems theory, which focus on the role of the environment and the task in the performance of functional activities.		
	Pin et al. 2019	SR	AMSTAR 2: very low	21 studies (10 RCT). GMFCS I-II, mean age over 4.8 yrs. ICP (interactive computer play) seemed to be more effective than conventional therapy in improving postural control and balance, with medium to large effect sizes for children with mild to moderate severity of CP. Future studies of high methodological rigour are required to verify the role of on-site guidance of the children during ICP and the effect on children with more severe CP.		
	Warnier et al. 2019	SR and meta-analysis	AMSTAR 2: high	26 studies, 9 RCT. GMFCS mostly I, one mixed, 6-18 yrs. VRT seems a promising intervention for rehabilitation in children with CP. The meta-analysis confirmed this positive effect. These results must be interpreted with caution due to differences in the interventions used, the lack of randomized-controlled trials, and the relatively small groups.		
	Ghai et al. 2019	SR	AMSTAR 2: low	14 RCT. 88% of the studies reported significant enhancements in gait performance after training with virtual reality. Meta-analyses revealed positive effects of virtual-reality training on gait velocity (Hedge's g = 0.68), stride length (0.30), cadence (0.66), and gross motor function measure (0.44). Subgroup analysis reported a training duration of 20–30min per session, ≤4 times per week across ≥8 weeks to allow maximum enhancements in gait velocity.		
	Novak et al. 2019	SR	AMSTAR 2: moderate	1 observational study. CP, 4-12 yrs. Virtual reality + biofeedback appeared to confer better balance than virtual reality alone.<br>1 RCT, 5 observational study. GMFCS I-III, 5-18 yrs. Wii Fit appeared to confer improved balance.		
	Albuquerque de Araujo et al. 2019	SR	AMSTAR 2: low	1 RCT. Spastic hemiplegic CP, mean age 9.6 yrs (SD 2.6), GMFCS I-II. Wii therapy and NDT, compared to NDT, improves PBS in short term (12 weeks).		
	Elbanna et al. 2019	SR	AMSTAR 2: low	rTMS improving upper limb function after intervention. tDCS improving balance and majority of gait variables. Not adverse effects. High satisfaction		**16.** Consider NIBS, combined with active-approaches, as one intervention to improve upper

NIBS (rTMS or tDCS)				levels for participants and caregivers. No long-term follow-up.	The panel discusses critical points: low evidence, short follow-up, limitations due to services' resources.	limb function, balance and gait in ambulatory patients, with caution relative to compliance of the child and local resources.<br><br>**WEAK +**
	Corsi et al. 2021	SR	AMSTAR 2: low	3 RCT relative to NIBS. Uni-bilateral CP, GMFCS I-III, 7-18 yrs. Vibratory platform, gait training, electrical stimulation, and transcranial stimulation were effective to improve spatiotemporal gait parameters, especially velocity in children with cerebral palsy.		
	Novak et al. 2019	SR	AMSTAR 2: moderate	4 SR relative to tDCS. Spastic or dystonic CP, 4-19 yrs. tDCS (0.7-1 mA) + a motor learning rehabilitation (treadmill or VR) appeared to confer improved gait velocity, stride length, cadence and balance compared to sham tDCS + rehabilitation. Safe and well tolerated by children. Adverse effects are rare, mild, and transient and include minor tingling, burning, itching, and skin redness.		
NMES	Management Of Cerebral Palsy In Children: A Guide For Allied Health Professionals (NSW Ministry of Health guidelines, 2018)	CPG	AGREE II: high	pAG.79 FES is widely used in adult stroke populations but can be used for children with cerebral palsy. There is emerging evidence to support the use of FES for children with cerebral palsy in the lower limb and inconclusive evidence for its use in the upper limb	The panel adapts the recommendation, considering SRs: poor data about adverse effects and compliance, low evidence, controversial results.	**17.** Future research is required to assess effectiveness of NMES in GMFCS I-III, in particular following botulinum injections or orthopaedic surgery.
	Salazar et al. 2019	SR	AMSTAR 2: high	6 RCT. Diplegic, hemiplegic, quadriplegic CP. Mean age 1.04-8.6 yrs. Low-quality of evidence suggests that NMES might be used as adjuvant therapy to improve gross motor function in children with spastic CP, particularly the sitting and standing dimensions of GMFM scale. Our results need to be carefully interpreted due to the small number of studies included and the reduced sample size in each study. Further research with adequate methodological quality, ample sample size, and long-term follow-up are still necessary.	Further research claimed.	
	Corsi et al. 2021	SR	AMSTAR 2: low	5 RCT relative to NMES. Uni-bilateral CP, GMFCS I-III, 7-18 yrs. Vibratory platform, gait training, electrical stimulation, and transcranial stimulation were effective to improve spatiotemporal gait parameters, especially velocity in children with cerebral palsy.	Low evidence.	

	Das et al. 2019	SR	AMSTAR 2: very low	34 SR. 0-18 yrs, uni-bilateral CP. Electrical stimulation is associated with fewer functional gains.	Poor functional gains.	
	Novak et al. 2019	SR	AMSTAR 2: moderate	5 SR. Mixed CP types, GMFCS I-IV, 1-19 yrs. Controversial results about improving gait, low-level evidence about improving standing and sitting.	Controversial results and low-level evidence.	
NDT	Management Of Cerebral Palsy In Children: A Guide For Allied Health Professionals (NSW Ministry of Health guidelines, 2018)	CPG	AGREE II: high	The child is a relatively passive recipient of the treatment, and the approach is embedded into the context of normal developmental sequence. Novak et al. (2013) report a lack of evidence to support the use of NDT in current practice and indicate that alternative evidence-based therapy interventions and approaches be used to provide more effective results.	The panel adopts the recommendation.	**18.** NDT is not recommended as intervention to improve gross motor function in CP children.<br><br>**STRONG -**
	Zanon et al. 2019	SR	AMSTAR 2: high	Included in Novak 2019, 3 RCT. CP diplegia, GMFCS I-III, 3-15 yrs. Further studies are required to assess the efficacy and safety of neurodevelopmental treatment for this purpose and until there, current evidence do not support its routinely use in practice.	Do not modify CPGs.	
	Das et al. 2019	SR	AMSTAR 2: very low	34 SR. 0-18 yrs, mostly hemiplegic CP. NDT has low-quality evidence	Do not modify CPGs.	
	Novak et al. 2019	SR	AMSTAR 2: moderate	1 SR, 4 RCT. GMFCS I-III, 2-15 yrs. the effectiveness of neurodevelopmental treatment for children with CP is unclear. Because of the lack of good certainty evidence, neurodevelopmental treatment should be used with caution. The child's response should be observed carefully. Recommendation downgraded to strong negative.	Do not modify CPGs.	
HIPPOTHE RAPY	Management Of Cerebral Palsy In Children: A Guide For Allied Health Professionals (NSW Ministry of Health guidelines, 2018)	CPG	AGREE II: high	PAG. 99 Horseback riding for therapy uses the horse's movement which has an individual and variable gait, tempo, rhythm, repetition, and cadence. It may influence neuromuscular development in humans. Improvements in trunk control and balance have been noted in children with cerebral palsy due to the physical adjustments to maintain proper alignment on the horse. From the current evidence it appears that hippotherapy and therapeutic horse riding have positive effects on balance and gross motor function in children with cerebral palsy although current literature and evidence is limited.	The panel adopt the recommendation, considering it as a complementary approach.	**19.** Consider horseback riding as a complementary activity, that might implement balance and trunk control, in GMFCS I-IV children. Considering cost-benefit balance and any individual risk factor is advisable before addressing to this approach. It must be differentiated from recreational horseback motor activity or adapted sport.

	De Guindos-Sanchez et al. 2020	SR and meta-analysis	AMSTAR 2: very low	10 RCT, mixed ages, GMFCS I-V. …effective to improve gross motor function: GMFM-66 total scores and GMFM-88 dimensions A, B, and E. Furthermore, positive effects have been showed on balance recovery and muscle spasticity reduction.	Do not modify CPGs.	**WEAK +**
	Novak et al. 2019	SR	AMSTAR 2: moderate	5 SR, 3 RCT. GMFCS I-V, uni-bilateral CP, 3-16 yrs. Conflicting findings in gross motor function. Hippotherapy conferred improved balance and posture, specifically improving trunk position and arm function in GMFCS I-IV.	Do not modify CPGs.	
	Albuquerque de Araujo et al. 2019	SR	AMSTAR 2: low	Just one low-level study dealed with hippotherapy (missing data about GMFCS, mean age 7 yrs, uni-bilateral CP). Very-low quality evidence suggests that BTI combined with other intervention enhances the effect of the other intervention alone on postural control at short-term in children and adolescents with CP.	Do not modify CPGs.	
SUIT THERAPY	Management Of Cerebral Palsy In Children: A Guide For Allied Health Professionals (NSW Ministry of Health guidelines, 2018)	CPG	AGREE II: high	PAG. 97 Currently there is conflicting and limited evidence on the benefits of suit therapy. Some studies have shown no improvement in motor function while other studies have shown some benefit, including improved gait parameters.<br>However, further investigation with larger sample sizes is recommended in the literature to determine the benefits of this intervention.	The panel adapt the recommendation due to conflicting evidence and adverse effects evidenced in the following studies.	**20.** The panel do not recommend suit therapy as a comprehensive approach, due to adverse effects reported.<br><br>STRONG -
	Novak et al. 2019	SR	AMSTAR 2: moderate	3 SR. CP mixed type, 3-17 yrs. … the suit might act on hip and shoulder stability and movement, given the suit is located over the hips and shoulders, whereas there was no effect on distal kinematics as the suit could not act on regions of the body not covered by the suit. Some children disliked wearing the suits and experienced adverse events including respiratory compromise, overheating and peripheral cyanosis. The suits also impeded function such as independent toileting and dressing.	Low evidence and several adverse effects.	
	Karadag-Saygi et al. 2019	SR	AMSTAR 2: low	29 studies (9 RCT). Studies were heterogenous in design, type of suit, size, study population, and outcomes measured. Some improvement reported in proximal stability, gross motor function, but low evidence and several adverse effects.	Low evidence and several adverse effects.	**21.** The panel recommends future research to verify effectiveness of suits as "functional orthoses" to enable or improve function.

TAPING	Inamdar et al. 2021	SR and meta-analysis	AMSTAR 2: high	12 RCT. Uni-bilateral CP, GMFCS I-V, 18 mo-puberty. Components of impairment remediation combined with functional balance training should be explored to improve sitting in children diagnosed with CP. Kinesio-taping may be an effective adjunct to conventional physical therapy in improving sitting ability in children with spastic bilateral CP. Given the benefits of early achievement of sitting, strong evidence-based research is needed.	Evidence as an adjunct approach.	**22.** Consider taping as an adjunct approach to physical therapy, as a functional orthosis, to improve gross motor or upper limb function. Contraindication: Contact dermatitis caused by tape<br><br>**WEAK +**
	Novak et al. 2019	SR	AMSTAR 2: moderate	7 SR. Uni-bilateral CP, GMFCS I-V, <18 yrs. Taping should be considered an adjunct to therapy, not stand-alone intervention, to improve gross motor and upper limbs function. Found to be most beneficial with GMFCS I-II, i.e. children with better selective motor control. Children had more active movement at the upper limbs, when the tape was elasticised compared to rigid tape. A small number of children will have a skin allergy to the tape, and allergy is a contraindication.	Evidence as an adjunct approach.	
ORTHOSES	Management Of Cerebral Palsy In Children: A Guide For Allied Health Professionals (NSW Ministry of Health guidelines, 2018)	CPG	AGREE II: high	PAG. 78 Functional orthoses generally position joints in a biomechanically advantageous position to either enable or improve function. Examples may include:<br>• Ankle foot orthoses (AFOs) – a variety of AFOs are available with varying purposes<br>• Wrist extension orthoses<br>• Neoprene wrist and thumb orthoses.<br>Positional orthoses aim to maintain corrected anatomical alignment of the joint and maintain range of motion around that joint. This may be important for ease of care, to reduce the requirement for future orthopaedic surgery and in some cases to maintain healthy skin integrity. Examples of positional orthoses may include:<br>• Spinal braces<br>• Leg or elbow wrap arounds<br>• Hip abduction orthoses.<br>The prescription and manufacture of upper and lower limb orthoses is common practice with children with cerebral palsy. Evidence suggests splints may be of some benefit when provided in conjunction with other therapies, although further research regarding splinting and orthoses is needed.	The panel adopt the recommendation.	**23.** Consider upper and lower limbs:<br>● positional orthoses (i.e., leg or elbow orthoses, spinal braces) to maintain corrected anatomical alignment or range of motion of the joint and/or skin integrity;<br>● functional orthoses (i.e., ankle-foot orthoses, wrist and thumb orthoses) to enable or improve function.<br><br>**STRONG +**
	Betancourt et al. 2019	SR	AMSTAR 2: very low	3 RCT, 14 prospective cohort studies. Uni-bilateral CO, GMFCS I-IV, 3-18 yrs. Children with cerebral palsy using ankle-foot orthoses had improved stride	Do not modify CPGs.	

				length and dorsiflexion angle during gait in a pooled meta-analyses of cohort studies and clinical trials.		
SERIAL CASTING	Management Of Cerebral Palsy In Children: A Guide For Allied Health Professionals (NSW Ministry of Health guidelines, 2018)	CPG	AGREE II: high	PAG. 78 Casting is a therapy intervention used to gain/restore muscle length and provide soft tissue elongation. Casting can be done as a one off or as a series of casts depending on the desired outcome and the child's tolerance for the cast. Casting is indicated when soft tissue contracture is interfering with function or causing potential biomechanical misalignment. Casting is not indicated when there are bony changes occurring at a joint. Casting only provides a short-term stretch and is usually required to be repeated at regular intervals and is particularly effective following Botulinum Toxin injections. There is currently no evidence to support upper limb casting being used in isolation, that is, it should be used in conjunction with other treatments that are focused on the activity level of the ICF.	The panel adopt the recommendation.	**24.** Consider casting (one or a series of casts depending on the desired outcome and the child's tolerance) following botulinum injection to provide short term stretch with the aim of improving dorsiflexion passive range of motion.<br><br>**STRONG +**
	Milne et al. 2020	SR	AMSTAR 2: low	25 studies (mixed type, mostly had poor methodological quality). Mixed population in 2 studies. Lower limb serial casting was found to be effective for: improving ankle dorsiflexion (DF) passive range of motion (PROM) in the immediate to short-term, decreasing hypertonicity measured by Modified Ashworth Scale (MAS) in the short-term. Serial casting with or without botulinum toxin type-A (BTX-A) did not significantly affect gross motor capacity measured by Gross Motor Function Measure (GMFM). Serial casting with pharmacological intervention achieved significantly more DF PROM than serial casting alone.	Do not modify CPGs.	
MASSAGE	Management Of Cerebral Palsy In Children: A Guide For Allied Health Professionals (NSW Ministry of Health guidelines, 2018)	CPG	AGREE II: high	PAG 96. Massage is considered one of a variety of complementary and alternative medicines. There are a wide variety of massage techniques from gentle effleurage to deep tissue massage or myofascial release. Use of massage may help relieve muscle pain and tightness with a flow on effect to improve gait, range of motion and/or balance. Massage<br>may be used to relax a child after a bath, before sleeping, or to prepare for a therapy session. Children and young adults with cerebral palsy may suffer from cramps and spasms, more than their non-cerebral palsy peers. There is little evidence on the benefits of massage in children with cerebral palsy even though it is often recommended for the	The panel agrees with considering the massage as a complementary and alternative medicine approach with low evidence of benefits, in terms of relaxation. Due to little evidence and its complementary role, no recommendation is provided.	/

				psychological and/or relaxation benefits due to changes seen in cortisol levels.		
	Guchan et al. 2020	SR	AMSTAR 2: very low	11 studies (7 RCT). Missing data relative to GMFCS in some studies, 0-18 yrs. Massage as an adjunct to traditional therapies should be used to reduce muscle tone in spastic-type CP.	Do not implement evidence.	
