# Supplementary material for: Care Pathways in Rehabilitation for Children and Adolescents with Cerebral Palsy: Distinctiveness of the Adaptation to the Italian Context
Source: Children (Basel). 2024 Jul 13;11(7):852. doi: 10.3390/children11070852 (PMC11275177; doi:10.3390/children11070852)
Supplement: Supplementary file 1 [file children-11-00852-s001.zip › Table S4.pdf]

Table S4. Evidence to Decision framework relative to Query 2 "What are the most effective motor rehabilitation approaches to improve gross motor or upper limb performance, in children and adolescents with CP?"

**Recommendation 1**<br>The motor rehabilitation approaches to improve gross motor or manual skills should consider the following issues:<br>- individualized active-use interventions;<br>- child-focused, age and developmentally appropriate goal to enhance motivation (i.e., playful activity or daily activity);<br>- the task should be analysed considering the child's skills as well as environmental limitations;<br>- consider not only motor skills but the child's multidimensional profile;<br>- consider the impact of the intervention on the child and the family;<br>- intervention might be structured with adaptations of the task and/or of the context (objects and environment), based on the analysis of the child's skills, to support motivation and avoid frustration;<br>- intervention should involve repetitive practice of task or part of it, without incurring burnout in the child;<br>- intensive interventions over short periods of time have generally been found to be more effective, time period in general resulted more effective, but the compliance of the child and of the family are to must be considered.			
Type of recommendation		Adopted x<br>Adapted □<br>De novo □	
FACTOR	DECISION		EXPLANATION
Balance of benefits	Benefits outweigh disadvantages x<br>Benefits and disadvantages are balanced □<br>Disadvantages outweigh benefits □		A comprehensive and individually tailored approach warranties an appropriate intervention.
Quality of the evidence	High x<br>Moderate □<br>Low □<br>Very low □		2 high quality GL, 1 low level quality GL and low-level to moderate level SR, confirmed by the expert opinions among the panel supported the recommendation.
Values and preferences	No significant variability x<br>Significant variability □		All agreed.
Resource required	Large costs □<br>Moderate x<br>Low □<br>Don't know □		An individually tailored and goal-oriented approach optimizes intervention costs.
Acceptability	Yes x<br>No □<br>Don't know □		The compliance of the child and of the family and the impact on them is considered.
Feasibility	Yes x<br>No □<br>Don't know □		Defining appropriate interventions reduces final costs.
Overall strength and direction of the recommendation	Strong x<br>Conditional □	Positive x<br>Negative □	

**Recommendation 2**<br>Consider bimanual interventions, performing functional tasks within enjoyable and playful activities, to improve bimanual skills in CP subjects. Bimanual interventions are intended as involving practising a specific task or goal, or parts of the task, focusing on the "activities" dimension rather than on "body and function". Consider the need for minimum cognitive skills to respond to cues, as a requirement for bimanual training.		
Type of recommendation	Adopted x	

		Adapted □<br>De novo □	
FACTOR	DECISION		EXPLANATION
Balance of benefits	Benefits outweigh disadvantages x<br>Benefits and disadvantages are balanced □<br>Disadvantages outweigh benefits □		Evidence demonstrated no disadvantage when addressing bimanual activities in playful context.
Quality of the evidence	High x<br>Moderate □<br>Low □<br>Very low □		High quality guidelines and low to moderate level SR, confirmed by the expert opinions among the panel supported the recommendation.
Values and preferences	No significant variability x<br>Significant variability □		All agreed.
Resource required	Large costs □<br>Moderate x<br>Low □<br>Don't know □		This approach is no more expensive than usual physical therapy.
Acceptability	Yes x<br>No □<br>Don't know □		Enjoyable and playful activities are advised, and child responsiveness is considered.
Feasibility	Yes x<br>No □<br>Don't know □		Feasible with appropriate information and training.
Overall strength and direction of the recommendation	Strong x<br>Conditional □	Positive x<br>Negative □	

**Recommendation 3**<br>Consider mCIMT combined with bimanual therapy in unilateral CP, to enhance manual skills. mCIMT might be applied up to two hours a day for a period of 2-10 weeks, performing functional tasks within enjoyable and playful activities. Consider reduced compliance and possible frustration, in particular with subjects with poorer function, as a limitation to applying mCIMT.			
Type of recommendation		Adopted □<br>Adapted x<br>De novo □	
FACTOR	DECISION		EXPLANATION
Balance of benefits	Benefits outweigh disadvantages x<br>Benefits and disadvantages are balanced □<br>Disadvantages outweigh benefits □		Evidence suggests considering mCIMT as an alternative approach to bimanual therapy.
Quality of the evidence	High x<br>Moderate □<br>Low □<br>Very low □		High quality guidelines and very low to moderate level SR, confirmed by the expert opinions among the panel supported the recommendation.
Values and preferences	No significant variability x<br>Significant variability □		All agreed.
Resource required	Large costs □<br>Moderate x<br>Low □<br>Don't know □		This approach is no more expensive than usual physical therapy.
Acceptability	Yes x<br>No □<br>Don't know □		The panel decided to point out reduced compliance and possible frustration differently from the literature, for subjects with poorer

			function, as limitations to applying mCIMT.
Feasibility	Yes x<br>No □<br>Don't know □		Feasible with appropriate information and training.
Overall strength and direction of the recommendation	Strong x<br>Conditional □	Positive x<br>Negative □	

**Recommendation 4**<br>Consider the following factors in the selection of mCIMT or bimanual intensive interventions:<br>- child/adolescent and family characteristics and preferences;<br>- therapist's expertise;<br>- costs of implementing the intervention;<br>- funding and service delivery models;<br>- resource availability.			
Type of recommendation		Adopted □<br>Adapted □<br>De novo x	
FACTOR	DECISION		EXPLANATION
Balance of benefits	Benefits outweigh disadvantages x<br>Benefits and disadvantages are balanced □<br>Disadvantages outweigh benefits □		Both bimanual therapy and m-CIMT are demonstrated to be effective.
Quality of the evidence	High x<br>Moderate □<br>Low □<br>Very low □		High quality SR with meta-analysis confirmed by the expert opinions among the panel supported the recommendation.
Values and preferences	No significant variability x<br>Significant variability □		All agreed.
Resource required	Large costs □<br>Moderate x<br>Low □<br>Don't know □		m-CIMT may be delivered by the parents, depending on the protocol, but requires therapist's monitoring. Bimanual therapy usually is delivered by the therapist. Both are recommended to be intensive. Therefore, final costs may be considered similar.
Acceptability	Yes x<br>No □<br>Don't know □		No disadvantages for the patients.
Feasibility	Yes x<br>No □<br>Don't know □		Feasible with appropriate training.
Overall strength and direction of the recommendation	Strong x<br>Conditional □	Positive x<br>Negative □	

**Recommendation 5**<br>Home programmes might be considered to increase the "dose" of therapy, to improve the performance of functional activities, depending on family and child compliance and based upon the following five step model:

a. Establish collaborative relationships between parents and therapist;<br>b. Set mutually agreed upon family and child goals;<br>c. Select therapeutic activities that focus on achieving family and child goals supported by best available evidence;<br>d. Support implementation of home programme through parent education, home visits and programme updates to sustain motivation;<br>e. Evaluate outcomes.			
Type of recommendation		Adopted □<br>Adapted x<br>De novo □	
FACTOR	DECISION		EXPLANATION
Balance of benefits	Benefits outweigh disadvantages □<br>Benefits and disadvantages are balanced x<br>Disadvantages outweigh benefits □		Home programmes may increase the "dose" of training, but requirements must be complied.
Quality of the evidence	High □<br>Moderate x<br>Low □<br>Very low □		High quality CPG suggest considering home programmes though they declare the evidence is limited (low to moderate).
Values and preferences	No significant variability x<br>Significant variability □		All agreed
Resource required	Large costs □<br>Moderate □<br>Low x<br>Don't know □		Entrust exercises to the family apparently reduces the costs of rehabilitation.
Acceptability	Yes □<br>No □<br>Don't know x		Compliance is one of the requirements.
Feasibility	Yes □<br>No □<br>Don't know x		Feasible with appropriate education of the parents, monitoring of the professionals, good compliance.
Overall strength and direction of the recommendation	Strong □<br>Conditional x	Positive x<br>Negative □	

**Recommendation 6**<br>AOT might be considered to improve bimanual performance in CP children, with particular attention to the severity of motor impairment and cognitive status as possible limitations.			
Type of recommendation		Adopted □<br>Adapted □<br>De novo x	
FACTOR	DECISION		EXPLANATION
Balance of benefits	Benefits outweigh disadvantages x<br>Benefits and disadvantages are balanced □<br>Disadvantages outweigh benefits □		Both bimanual therapy and m-CIMT are demonstrated to be effective.
Quality of the evidence	High □<br>Moderate x<br>Low □<br>Very low □		Two SR (low and moderate level of evidence) were in favour of AOT. Nonetheless, one high level SR with meta-analysis, concluded that no evidence of benefit had been found to draw a firm conclusion regarding the effectiveness of AOT in the rehabilitation of children with CP due to limitations in methodological

			quality and variations between studies.
Values and preferences	No significant variability x<br>Significant variability □		All agreed.
Resource required	Large costs □<br>Moderate x<br>Low □<br>Don’t know □		Costs depend on the type of technology involved while delivering AOT.
Acceptability	Yes □<br>No □<br>Don’t know x		Attention must be paid with CP children depending on the severity of motor impairment and cognitive status; further studies are needed to determine the optimal frequency, intensity, and time of AOT on these particular study subjects.
Feasibility	Yes x<br>No □<br>Don’t know □		Feasible with appropriate training.
Overall strength and direction of the recommendation	Strong □<br>Conditional x	Positive x<br>Negative □	

**Recommendation 7**<br>HABIT-ILE might be considered to improve upper and lower limb motor function in GMFCS I-IV subject age 6-16 yrs.			
Type of recommendation		Adopted □<br>Adapted □<br>De novo x	
FACTOR	DECISION		EXPLANATION
Balance of benefits	Benefits outweigh disadvantages □<br>Benefits and disadvantages are balanced x<br>Disadvantages outweigh benefits □		Low-level evidence support HABIT_ILE in camp format, compared to usual care, but limitations and benefits need to be further enquired.
Quality of the evidence	High □<br>Moderate □<br>Low x<br>Very low □		Only one SR.
Values and preferences	No significant variability x<br>Significant variability □		All agreed.
Resource required	Large costs x<br>Moderate □<br>Low □<br>Don’t know □		Costs relative to camp format might be larger than usual therapy.
Acceptability	Yes x<br>No □<br>Don’t know □		A camp, group and playing context is engaging for children.
Feasibility	Yes □<br>No □<br>Don’t know x		Too limited experience.
Overall strength and direction of the recommendation	Strong □<br>Conditional x	Positive x<br>Negative □	

**Recommendation 8**<br>Provide an adapted physical therapy programme following treatment with botulinum toxin type A, continuous pump-administered intrathecal baclofen, orthopaedic surgery or selective dorsal rhizotomy.			
Type of recommendation		Adopted x<br>Adapted □<br>De novo □	
FACTOR	DECISION		EXPLANATION
Balance of benefits	Benefits outweigh disadvantages x<br>Benefits and disadvantages are balanced □<br>Disadvantages outweigh benefits □		
Quality of the evidence	High x<br>Moderate □<br>Low □<br>Very low □		High-level CPG.
Values and preferences	No significant variability x<br>Significant variability □		All agreed.
Resource required	Large costs □<br>Moderate x<br>Low □<br>Don't know □		Costs are necessary to implement treatment.
Acceptability	Yes x<br>No □<br>Don't know □		
Feasibility	Yes x<br>No □<br>Don't know □		Depending on the training.
Overall strength and direction of the recommendation	Strong x<br>Conditional □	Positive x<br>Negative □	

**Recommendation 9**<br>Consider task-specific, intensive, and child-initiated intervention to achieve or re-achieve after an intervention (i.e., orthopaedic surgery) gross motor skills such as sitting, standing, balance and gait. Equipment and orthoses may be utilised to assist in maintaining the person's appropriate posture and movement.			
Type of recommendation		Adopted □<br>Adapted x<br>De novo □	
FACTOR	DECISION		EXPLANATION
Balance of benefits	Benefits outweigh disadvantages x<br>Benefits and disadvantages are balanced □<br>Disadvantages outweigh benefits □		
Quality of the evidence	High x<br>Moderate □<br>Low □<br>Very low □		High-level CPG and very low to high-level SR.
Values and preferences	No significant variability x<br>Significant variability □		All agreed.
Resource required	Large costs □<br>Moderate x<br>Low □<br>Don't know □		Costs are necessary to implement treatment.

Acceptability	Yes x<br>No □<br>Don't know □		
Feasibility	Yes x<br>No □<br>Don't know □		Depending on the training.
Overall strength and direction of the recommendation	Strong x<br>Conditional □	Positive x<br>Negative □	

**Recommendation 10**<br>As for subjects with typical development, reducing sedentary behaviour and encouraging light intensity activities throughout the day as fitness training (i.e., gross motor activity training, cycling, overground or treadmill walking, modified sports) should be integrated into daily life of CP subjects, with sufficient motor skills to be able to undertake training. Consider that fitness training may provide short-term benefits, in terms of gross-motor function and aerobic fitness, but these are not maintained when training stops			
Type of recommendation		Adopted x<br>Adapted □<br>De novo □	
FACTOR	DECISION		EXPLANATION
Balance of benefits	Benefits outweigh disadvantages x<br>Benefits and disadvantages are balanced □<br>Disadvantages outweigh benefits □		
Quality of the evidence	High x<br>Moderate □<br>Low □<br>Very low □		High-level CPG and moderate to high-level SR.
Values and preferences	No significant variability x<br>Significant variability □		All agreed.
Resource required	Large costs □<br>Moderate □<br>Low □<br>Don't know x		These activities are considered leisure activities, rather than rehabilitative interventions.
Acceptability	Yes x<br>No □<br>Don't know □		
Feasibility	Yes □<br>No □<br>Don't know x		Depending on the context, socio-economic family level.
Overall strength and direction of the recommendation	Strong x<br>Conditional □	Positive x<br>Negative □	

**Recommendation 11**<br>Strengthening training for lower limbs, is an accepted intervention for CP children with sufficient motor skills (i.e., selective motor control) to be able to undertake training, with the only objective of improving muscle strength. It requires a small number of repetitions until fatigue, rest periods, short duration and low frequency. Strengthening training does not improve motor function.			
Type of recommendation		Adopted □<br>Adapted x<br>De novo □	
FACTOR	DECISION		EXPLANATION
Balance of benefits	Benefits outweigh disadvantages □		

<table>
<tr><td></td><td colspan="2">Benefits and disadvantages are balanced x
Disadvantages outweigh benefits □</td><td></td></tr>
<tr><td>Quality of the evidence</td><td colspan="2">High □
Moderate x
Low □
Very low □</td><td>CPGs suggest it to improve lower limb muscle strength but declare no benefits in gross motor function.</td></tr>
<tr><td>Values and preferences</td><td colspan="2">No significant variability x
Significant variability □</td><td>All agreed.</td></tr>
<tr><td>Resource required</td><td colspan="2">Large costs □
Moderate x
Low □
Don’t know □</td><td>Costs are necessary to implement treatment, but it must be clear that benefits are limited to muscle strength and in the short term.</td></tr>
<tr><td>Acceptability</td><td colspan="2">Yes x
No □
Don’t know □</td><td>For CP children with sufficient motor skills (i.e., selective motor control) to be able to undertake training.</td></tr>
<tr><td>Feasibility</td><td colspan="2">Yes x
No □
Don’t know □</td><td>Depending on the training.</td></tr>
<tr><td>Overall strenght and direction of the recommendation</td><td>Strong □
Conditional x</td><td>Positive x
Negative □</td><td></td></tr>
</table>

<table>
<tr><td colspan="4">Recommendation 12
Consider hydrotherapy as a complementary approach in rehabilitation programmes, to recover gross motor function following orthopaedic surgery, to improve fitness and endurance. It must be differentiated from recreational motor activity into the water or adapted sport, which is advisable, whenever possible, to improve fitness. Possible limitations: open wounds, child’s compliance, contextual barriers, and services’ resources.</td></tr>
<tr><td colspan="2">Type of recommendation</td><td colspan="2">Adopted □
Adapted x
De novo □</td></tr>
<tr><td>FACTOR</td><td colspan="2">DECISION</td><td>EXPLANATION</td></tr>
<tr><td>Balance of benefits</td><td colspan="2">Benefits outweigh disadvantages x
Benefits and disadvantages are balanced □
Disadvantages outweigh benefits □</td><td></td></tr>
<tr><td>Quality of the evidence</td><td colspan="2">High □
Moderate x
Low □
Very low □</td><td>One CPG and two very low and moderate-level SR</td></tr>
<tr><td>Values and preferences</td><td colspan="2">No significant variability x
Significant variability □</td><td>All agreed</td></tr>
<tr><td>Resource required</td><td colspan="2">Large costs □
Moderate x
Low □
Don’t know □</td><td>Contextual barriers and services’ resources may limit.</td></tr>
<tr><td>Acceptability</td><td colspan="2">Yes x
No □
Don’t know □</td><td>Open wounds, child’s compliance are limitations</td></tr>
<tr><td>Feasibility</td><td colspan="2">Yes x
No □
Don’t know □</td><td>Depending on the training and contextual barriers.</td></tr>
<tr><td>Overall strength and direction of the recommendation</td><td>Strong □
Conditional x</td><td>Positive x
Negative □</td><td></td></tr>
</table>

Recommendation 13

Consider treadmill training, with or without body weight support, as one possible alternative approach to overground walking, to improve gait endurance, gait speed and gross motor function. Being just one type of gait training approach, consider resources and preferences of subjects and their families, and of service providers.

Future research is required to define need and parameters of body-weight support and motion guidance, for mechanically assisted walking devices.

Type of recommendation		Adopted x<br>Adapted □<br>De novo □	
FACTOR	DECISION		EXPLANATION
Balance of benefits	Benefits outweigh disadvantages □<br>Benefits and disadvantages are balanced x<br>Disadvantages outweigh benefits □		
Quality of the evidence	High □<br>Moderate x<br>Low □<br>Very low □		One CPG and two moderate to high-level SRs.
Values and preferences	No significant variability x<br>Significant variability □		All agreed.
Resource required	Large costs □<br>Moderate x<br>Low □<br>Don't know □		Contextual barriers and services' resources may limit.
Acceptability	Yes x<br>No □<br>Don't know □		Child's compliance may limit.
Feasibility	Yes x<br>No □<br>Don't know □		Depending on the training services' resources.
Overall strength and direction of the recommendation	Strong □<br>Conditional x	Positive x<br>Negative □	

Recommendation 14

Consider virtual reality in terms of videogames to improve hand function in an individualized rehabilitation programme, as a complement to conventional therapies. Possible limitations: child's compliance, contextual barriers and resources.

Type of recommendation		Adopted □<br>Adapted □<br>De novo x	
FACTOR	DECISION		EXPLANATION
Balance of benefits	Benefits outweigh disadvantages □<br>Benefits and disadvantages are balanced x<br>Disadvantages outweigh benefits □		Complementary to usual direct treatment.
Quality of the evidence	High □<br>Moderate x<br>Low □<br>Very low □		Low to moderate-level SRs.
Values and preferences	No significant variability x		All agreed.

	Significant variability □		
Resource required	Large costs □<br>Moderate x<br>Low □<br>Don't know □		Family and/or services' resources may limit.
Acceptability	Yes x<br>No □<br>Don't know □		Child's compliance may limit, but usually engaged by VR games.
Feasibility	Yes x<br>No □<br>Don't know □		Depending on the training, preferences, and services' resources.
Overall strength and direction of the recommendation	Strong □<br>Conditional x	Positive x<br>Negative □	

**Recommendation 15**<br>Consider virtual reality games (i.e., Nintendo WII Balance) as an additional treatment for improving functional and dynamic balance in children with CP at GMFCS level I-IV, combined with conventional physical therapy. Possible limitations: child's compliance, contextual barriers and resources.		
Type of recommendation	Adopted □<br>Adapted □<br>De novo x	
FACTOR	DECISION	EXPLANATION
Balance of benefits	Benefits outweigh disadvantages □<br>Benefits and disadvantages are balanced x<br>Disadvantages outweigh benefits □	Complementary to usual direct treatment.
Quality of the evidence	High □<br>Moderate x<br>Low □<br>Very low □	Very low to moderate-level SRs.
Values and preferences	No significant variability x<br>Significant variability □	All agreed.
Resource required	Large costs □<br>Moderate x<br>Low □<br>Don't know □	Family and/or services' resources may limit.
Acceptability	Yes x<br>No □<br>Don't know □	Child's compliance may limit, but usually engaged by VR games.
Feasibility	Yes x<br>No □<br>Don't know □	Depending on the training, preferences, and services' resources.
Overall strength and direction of the recommendation	Strong □<br>Conditional x / Positive x<br>Negative □	

**Recommendation 16**<br>Consider NIBS, combined with active-approaches, as one intervention, to improve upper limb function, balance, and gait in ambulatory patients, with caution relative to compliance of the child and local resources.		
Type of recommendation	Adopted □<br>Adapted □<br>De novo x	
FACTOR	DECISION	EXPLANATION

Balance of benefits	Benefits outweigh disadvantages □<br>Benefits and disadvantages are balanced x<br>Disadvantages outweigh benefits □		Complementary to usual direct treatment.
Quality of the evidence	High □<br>Moderate x<br>Low □<br>Very low □		Low to moderate-level SRs.
Values and preferences	No significant variability x<br>Significant variability □		All agreed.
Resource required	Large costs x<br>Moderate □<br>Low □<br>Don't know □		Services' resources may limit.
Acceptability	Yes x<br>No □<br>Don't know □		Child's compliance may limit.
Feasibility	Yes x<br>No □<br>Don't know □		Depending on the training and services' resources.
Overall strength and direction of the recommendation	Strong □<br>Conditional x	Positive x<br>Negative □	

**Recommendation 17**<br>Future research is required to assess effectiveness of NMES in GMFCS I-III, in particular following botulinum injections or orthopaedic surgery.			
Type of recommendation		Adopted □<br>Adapted x<br>De novo □	
FACTOR	DECISION		EXPLANATION
Balance of benefits	Benefits outweigh disadvantages □<br>Benefits and disadvantages are balanced x<br>Disadvantages outweigh benefits □		Complementary to usual direct treatment.
Quality of the evidence	High □<br>Moderate □<br>Low x<br>Very low □		One CPG and very low to moderate-level SRs.
Values and preferences	No significant variability x<br>Significant variability □		All agreed.
Resource required	Large costs x<br>Moderate □<br>Low □<br>Don't know □		Services' resources may limit.
Acceptability	Yes x<br>No □<br>Don't know □		Child's compliance may limit.
Feasibility	Yes x<br>No □<br>Don't know □		Depending on the training and services' resources.
Overall strength and direction of the recommendation	Strong □<br>Conditional □	Positive □<br>Negative □	Research recommendation.

<table>
<tr><td colspan="4">Recommendation 18
NDT is not recommended as intervention to improve gross motor function in CP children.</td></tr>
<tr><td colspan="2">Type of recommendation</td><td colspan="2">Adopted x
Adapted □
De novo □</td></tr>
<tr><td>FACTOR</td><td colspan="2">DECISION</td><td>EXPLANATION</td></tr>
<tr><td>Balance of benefits</td><td colspan="2">Benefits outweigh disadvantages □
Benefits and disadvantages are balanced □
Disadvantages outweigh benefits x</td><td>No benefits demonstrated on gross motor function and passive treatment.</td></tr>
<tr><td>Quality of the evidence</td><td colspan="2">High □
Moderate □
Low □
Very low x</td><td>High-level CPG and very low to high-level SRs report a lack of evidence to support the use of NDT.</td></tr>
<tr><td>Values and preferences</td><td colspan="2">No significant variability x
Significant variability □</td><td>All agreed.</td></tr>
<tr><td>Resource required</td><td colspan="2">Large costs □
Moderate x
Low □
Don't know □</td><td>Costs are similar to other direct physiotherapic treatment.</td></tr>
<tr><td>Acceptability</td><td colspan="2">Yes □
No □
Don't know x</td><td>Child's compliance may limit.</td></tr>
<tr><td>Feasibility</td><td colspan="2">Yes x
No □
Don't know □</td><td>Depending on the training.</td></tr>
<tr><td>Overall strength and direction of the recommendation</td><td>Strong x
Conditional □</td><td>Positive □
Negative x</td><td></td></tr>
</table>

<table>
<tr><td colspan="4">Recommendation 19
Consider horseback riding as a complementary activity, that might implement balance and trunk control, in GMFCS I-IV children. Considering cost-benefit balance and any individual risk factor is advisable before addressing patients to this approach. It must be differentiated from recreational horseback motor activity or adapted sport.</td></tr>
<tr><td colspan="2">Type of recommendation</td><td colspan="2">Adopted x
Adapted □
De novo □</td></tr>
<tr><td>FACTOR</td><td colspan="2">DECISION</td><td>EXPLANATION</td></tr>
<tr><td>Balance of benefits</td><td colspan="2">Benefits outweigh disadvantages □
Benefits and disadvantages are balanced x
Disadvantages outweigh benefits □</td><td>Complementary to usual treatment.</td></tr>
<tr><td>Quality of the evidence</td><td colspan="2">High □
Moderate x
Low □
Very low □</td><td>High-level CPG and very low to moderate-level SRs.</td></tr>
<tr><td>Values and preferences</td><td colspan="2">No significant variability x
Significant variability □</td><td>All agreed.</td></tr>
<tr><td>Resource required</td><td colspan="2">Large costs x
Moderate □
Low □
Don't know □</td><td>Services' resources may limit.</td></tr>
<tr><td>Acceptability</td><td colspan="2">Yes x</td><td>Child's compliance may limit.</td></tr>
</table>

	No □<br>Don't know □		
Feasibility	Yes x<br>No □<br>Don't know □		Depending on the training and services' resources.
Overall strength and direction of the recommendation	Strong □<br>Conditional x	Positive x<br>Negative □	

**Recommendation 20**<br>The panel do not recommend suit therapy as a comprehensive approach, due to adverse effects reported.			
Type of recommendation		Adopted □<br>Adapted x<br>De novo □	
FACTOR	DECISION		EXPLANATION
Balance of benefits	Benefits outweigh disadvantages □<br>Benefits and disadvantages are balanced □<br>Disadvantages outweigh benefits x		Conflicting evidence and adverse effects evidenced in the SRs.
Quality of the evidence	High □<br>Moderate x<br>Low □<br>Very low □		High-level CPG and low to moderate-level SRs.
Values and preferences	No significant variability x<br>Significant variability □		All agreed.
Resource required	Large costs □<br>Moderate x<br>Low □<br>Don't know □		Depending on the type of suit and comprehensive approach.
Acceptability	Yes □<br>No x<br>Don't know □		Child's compliance and reported adverse effects are limitations.
Feasibility	Yes □<br>No □<br>Don't know x		
Overall strength and direction of the recommendation	Strong x<br>Conditional □	Positive □<br>Negative x	

**Recommendation 21**<br>The panel recommend future research to verify effectiveness of suits as "functional orthoses" to enable or improve function.			
Type of recommendation		Adopted □<br>Adapted □<br>De novo x	
FACTOR	DECISION		EXPLANATION
Balance of benefits	Benefits outweigh disadvantages □<br>Benefits and disadvantages are balanced x<br>Disadvantages outweigh benefits □		Complementary to usual direct treatment as a functional orthosis.
Quality of the evidence	High □<br>Moderate x<br>Low □<br>Very low □		High-level CPG and low to moderate-level SRs.

Values and preferences	No significant variability x<br>Significant variability □		All agreed.
Resource required	Large costs □<br>Moderate x<br>Low □<br>Don't know □		As other orthoses.
Acceptability	Yes x<br>No □<br>Don't know □		Child's compliance may limit.
Feasibility	Yes x<br>No □<br>Don't know □		Depending on the training.
Overall strength and direction of the recommendation	Strong □<br>Conditional □	Positive □<br>Negative □	Research recommendation.

**Recommendation 22**<br>Consider taping as an adjunct approach to physical therapy, as a functional orthosis, to improve gross motor or upper limb function. Contraindication: Contact dermatitis caused by tape.			
Type of recommendation		Adopted □<br>Adapted □<br>De novo x	
FACTOR	DECISION		EXPLANATION
Balance of benefits	Benefits outweigh disadvantages x<br>Benefits and disadvantages are balanced □<br>Disadvantages outweigh benefits □		Complementary to usual treatment.
Quality of the evidence	High □<br>Moderate x<br>Low □<br>Very low □		Moderate to high-level SRs .
Values and preferences	No significant variability x<br>Significant variability □		All agreed.
Resource required	Large costs □<br>Moderate □<br>Low x<br>Don't know □		
Acceptability	Yes x<br>No □<br>Don't know □		Skin irritation and child's compliance may limit.
Feasibility	Yes x<br>No □<br>Don't know □		Depending on the training.
Overall strength and direction of the recommendation	Strong □<br>Conditional x	Positive x<br>Negative □	

**Recommendation 23**<br>Consider upper and lower limbs:<br>● positional orthoses (i.e., leg or elbow orthoses, spinal braces) to maintain corrected anatomical alignment or range of motion of the joint and/or skin integrity;<br>● functional orthoses (i.e., ankle-foot orthoses, wrist and thumb orthoses) to enable or improve function.	
Type of recommendation	Adopted x

		Adapted □<br>De novo □	
FACTOR	DECISION		EXPLANATION
Balance of benefits	Benefits outweigh disadvantages x<br>Benefits and disadvantages are balanced □<br>Disadvantages outweigh benefits □		
Quality of the evidence	High □<br>Moderate x<br>Low □<br>Very low □		High-level CPG and experts' opinion support the use of orthoses even though the evidence is limited.
Values and preferences	No significant variability x<br>Significant variability □		All agreed.
Resource required	Large costs □<br>Moderate x<br>Low □<br>Don't know □		Costs are necessary to implement treatment.
Acceptability	Yes x<br>No □<br>Don't know □		Compliance of the patient may limit mostly because of aesthetics and social participation.
Feasibility	Yes x<br>No □<br>Don't know □		Depending on the expertise of the orthotist.
Overall strength and direction of the recommendation	Strong x<br>Conditional □	Positive x<br>Negative □	

**Recommendation 24**<br>Consider casting (one or a series of casts depending on the desired outcome and the child's tolerance) following botulinum injection to provide short term stretch with the aim of improving dorsiflexion passive range of motion.			
Type of recommendation		Adopted x<br>Adapted □<br>De novo □	
FACTOR	DECISION		EXPLANATION
Balance of benefits	Benefits outweigh disadvantages x<br>Benefits and disadvantages are balanced □<br>Disadvantages outweigh benefits □		
Quality of the evidence	High □<br>Moderate x<br>Low □<br>Very low □		High-level CPG and experts' opinion support the use of casting limited to ankle joint.
Values and preferences	No significant variability x<br>Significant variability □		All agreed.
Resource required	Large costs □<br>Moderate □<br>Low x<br>Don't know □		Costs are necessary to implement treatment.
Acceptability	Yes x<br>No □<br>Don't know □		Compliance of the patient may limit. Not indicated in case of fixed contractures.
Feasibility	Yes x<br>No □		Depending on the training.

	Don’t know □		
Overall strength and direction of the recommendation	Strong x<br>Conditional □	Positive x<br>Negative □	
